# Effect of naltrexone pretreatment on ketamine-induced glutamatergic activity and symptoms of depression: a randomized crossover study

**DOI:** 10.1038/s41591-025-03800-w

**Published:** 2025-07-24

**Authors:** Luke A. Jelen, David J. Lythgoe, James M. Stone, Allan H. Young, Mitul A. Mehta

**Affiliations:** 1 The Institute of Psychiatry, Psychology and Neuroscience, King’s College London, London, UK; 2 South London and Maudsley NHS Foundation Trust, London, UK; 3 Brighton and Sussex Medical School (BSMS), University of Sussex, Brighton, UK; 4 Sussex Partnership NHS Foundation Trust, Worthing, UK; 5 Imperial College London, London, UK

**Keywords:** Depression, Molecular neuroscience

## Abstract

We investigated the potential role of the opioid system in modulating glutamatergic effects of ketamine administration in major depressive disorder. Twenty-six adults with major depressive disorder participated in a double-blind crossover study, receiving oral placebo or 50 mg naltrexone before an intravenous infusion of 0.5 mg per kg ketamine. Brain glutamatergic activity in the anterior cingulate cortex was measured using functional magnetic resonance spectroscopy and depressive symptoms were assessed with the Montgomery–Åsberg Depression Rating Scale. Naltrexone attenuated the increase in glutamate + glutamine to total *N*-acetylaspartate ratio during ketamine infusion compared to placebo (*F*_1,253_ = 4.83, *P* = 0.029) and also attenuated the reduction in Montgomery–Åsberg Depression Rating Scale scores on day 1 (condition-by-time interaction, *F*_1,74 _= 5.39, *P* = 0.023). These findings demonstrate that the opioid system modulates the acute response to ketamine and subsequent antidepressant effects. Interactions between the glutamate and opioid systems may have implications for the development of new depression treatment strategies. ClinicalTrials.gov registration: NCT04977674.

## Abstract (web summary)

The opioid system modulates ketamine’s effects in major depressive disorder, with naltrexone reducing acute glutamatergic activity and acute antidepressant effects.

## Main

Ketamine, a dissociative anesthetic and uncompetitive *N*-methyl-d-aspartate (NMDA) receptor antagonist, has emerged as a new treatment for major depressive disorder (MDD)^1^. Reductions in depressive symptoms have been demonstrated following a single subanesthetic ketamine infusion, including in cases of treatment-resistant depression^2^.

Although the specific mechanisms underpinning ketamine’s antidepressant actions are yet to be fully determined, one hypothesis proposes that ketamine preferentially blocks NMDA receptors expressed on γ-aminobutyric acid (GABA) interneurons, leading to disinhibition of pyramidal neurons and increased glutamate release^3^. This so-called glutamate surge stimulates postsynaptic α-amino-3-hydroxy-5-methyl-4-isoxazole propionic acid (AMPA) receptors leading to activation of downstream neuroplastic signaling pathways and synaptogenesis^4^.

Supporting this hypothesis, increases in glutamatergic compounds have been observed following ketamine administration in the medial prefrontal cortex (mPFC) in studies with rodents^5–7^. In humans, using proton magnetic resonance spectroscopy (^1^H-MRS), ketamine administration has been found to acutely increase anterior cingulate cortex (ACC) glutamate^8^, glutamine^9^ and glutamate + glutamine (Glx)^10^ in healthy volunteers, and to increase Glx in the mPFC in patients with MDD^11^; however, this finding has not been consistent^12^. Further work, utilizing ^13^C-MRS, recorded a rapid increase in ^13^C-glutamine enrichment early during ketamine infusion in healthy controls and patients with MDD, indicating an acute surge in prefrontal glutamate–glutamine cycling^13^. Twenty-four hours after ketamine administration, around the time of peak antidepressant response, a ^1^H-MRS study in MDD found no significant changes in glutamatergic measures in the ACC^14^. Together, these findings support the theory that subanesthetic doses of ketamine acutely increase glutamatergic activity but it is not clear if this is necessary for the antidepressant response.

Beyond the glutamate system, ketamine interacts with a range of other sites, including opioid receptors, albeit with lower affinity than with the NMDA receptor^15,16^. Preclinical work has demonstrated that activation of mu opioid receptors (MORs) is “necessary, but not sufficientˮ for ketamine to reduce depressive-like behaviors^17^, that (*S*)-ketamine involves direct interactions with brain MORs at doses and concentrations relevant to its reinforcement and antidepressant-like efficacy^18^, and that β-endorphin presence and opioid receptor activation in the mPFC may be required for ketamine’s antidepressant actions^19^. In a recent double-blind crossover clinical study, pretreatment with the opioid receptor antagonist naltrexone significantly attenuated the antidepressant and antisuicidal effects of a single ketamine dose in 12 adults with treatment-resistant depression^20,21^, suggesting opioid system activation may be required for ketamine’s antidepressant effects. An uncontrolled case series of five individuals with depression and alcohol-use disorder did not replicate these findings^22^. Importantly, these clinical studies have been limited by small sample sizes and did not explore mechanisms underlying any potential opioid-mediated effects.

A key question is whether opioid antagonism suppresses ketamine-induced glutamatergic activity in individuals with depression. Considering the coexpression of mu opioid and NMDA receptors^23,24^, it is feasible that glutamate release is affected by opioid modulation. Using standard ^1^H-MRS to investigate this is limited in terms of temporal resolution. Functional ^1^H-MRS (^1^H-fMRS) is a technique with superior temporal characteristics that enables the dynamic monitoring of glutamatergic metabolites^25,26^ and has previously been applied to measure anterior cingulate glutamatergic dynamics in patient groups^27,28^. In this study, we sought to test the hypothesis that ketamine administration in patients with depression leads to an acute increase in glutamatergic activity in the ACC, as measured by ^1^H-fMRS, and that pretreatment with the opioid receptor antagonist naltrexone attenuates this increase. We further tested whether pretreatment with naltrexone attenuates the acute antidepressant effects of ketamine.

## Results

### Participants

From the 28 participants randomized, 26 participants completed the crossover and underwent both the placebo-plus-ketamine and naltrexone-plus-ketamine arms of the study (Supplementary Fig. 1). The interval between the two study arm visits ranged from 14 to 33 days (mean = 19.11, s.d. = 4.32 days). Participant demographics and clinical characteristics are shown in Table 1. Regular psychotropic treatment received during the study is shown in Supplementary Table 1.

**Table 1:** Demographic and clinical characteristics

Characteristic		Overall
Age, years (mean (s.d.))		35.08 (7.50)
Sex, no. (%)	Female	13 (50.0)
	Male	13 (50.0)
Race/ethnicity, no. (%)	Asian/Asian British—Indian	2 (7.7)
	Asian/Asian British—Other	3 (11.5)
	Black/Black British—African	2 (7.7)
	Black/Black British—Caribbean	1 (3.8)
	White Irish	2 (7.7)
	White Other	4 (15.4)
	White UK	12 (46.2)
Employed, no. (%)	Yes	17 (65.4)
	No	6 (23.1)
	Student	3 (11.5)
BMI, kg per m^2^ (mean (s.d.))		23.85 (2.72)
Age at first MDD onset, years (mean (s.d.))		22.65 (8.28)
Duration of current MDD episode, years (median (IQR))		3.25 (2.62, 11.75)
Recurrent MDD, no. (%)	Yes	12 (46.2)
	No	14 (53.8)
Number of antidepressants trialed, no. (%)	1 to 2	16 (61.5)
	3 to 4	6 (23.1)
	5 to 6	3 (11.5)
	7–10	1 (3.8)
Psychological therapy trialed, no. (%)	Yes	26 (100)
Current antidepressant treatment, no. (%)	Yes	12 (46.2)
	No	14 (53.8)
HAM-D at screening (mean (s.d.))		21.65 (2.73)

IQR, interquartile range.

**Fig. 1:**
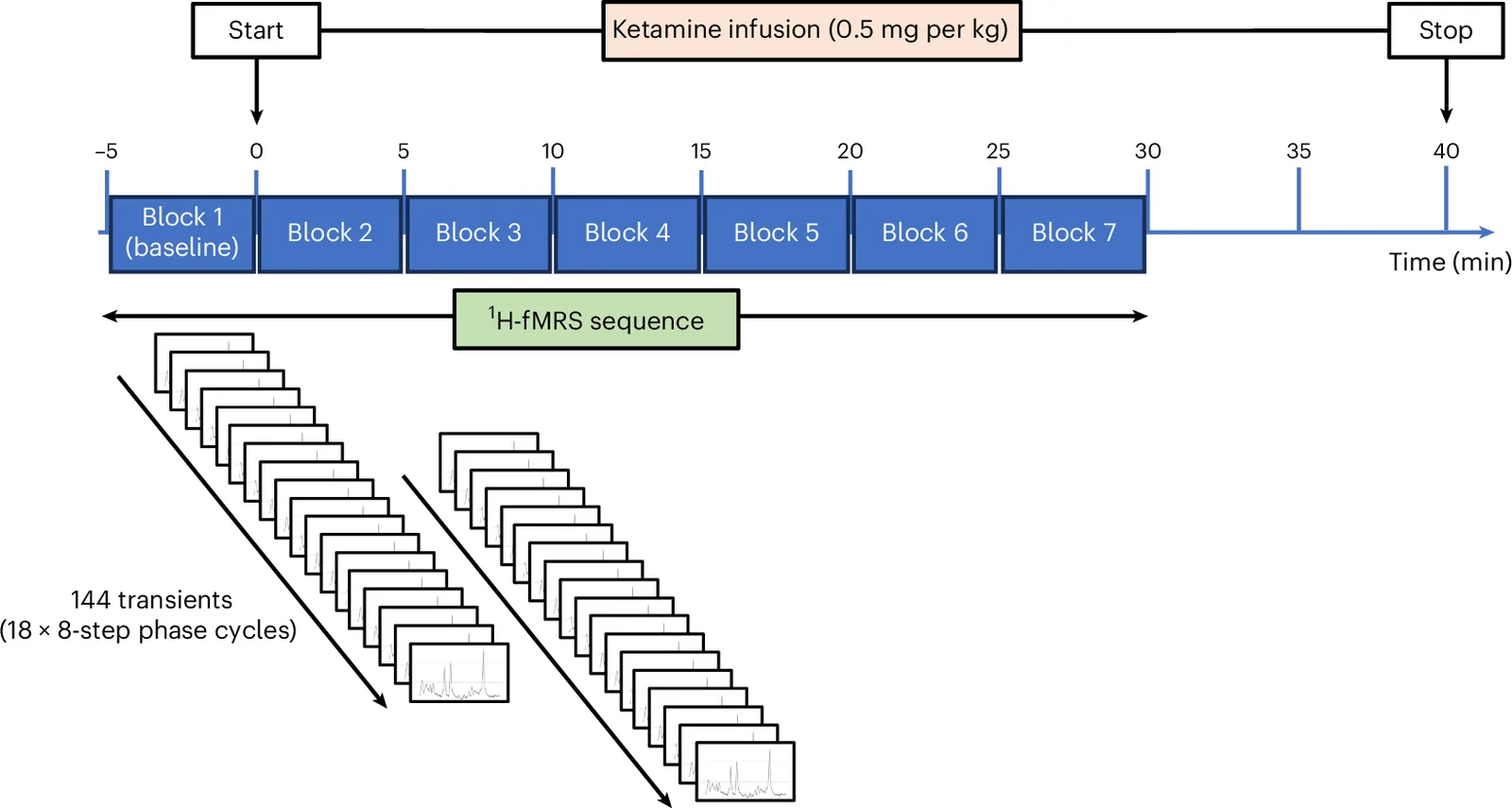
^1^H-fMRS experimental overview. Spectra were acquired continuously for a 5-min baseline period and during the initial 30 min of the ketamine infusion using point-resolved spectroscopy with chemical selective suppression for water suppression (repetition time (TR) = 2,000 ms, echo time (TE) = 40 ms, 8-step phase cycle, 1,040 transients). Each block consisted of 144 transients (18 × 8-step phase cycles). A total of 16 water unsuppressed transients (2 × 8-step phase cycles) were acquired at the end of the sequence.

To test the main hypothesis that naltrexone attenuates the effects of ketamine in individuals with depression, we assessed clinical symptoms, subjective ratings and an imaging-based measure of glutamatergic metabolites using ^1^H-fMRS. Spectra were acquired continuously for a 5-min baseline period and throughout the first 30 min of the ketamine infusion (Fig. 1).

### Clinical and subjective measures

#### Depression

Depressive symptoms were assessed with the clinician-rated Montgomery–Asberg Depression Rating Scale (MADRS) as the primary measure, together with the self-report Quick Inventory of Depressive Symptomatology–Self Report (QIDS–SR) and the Maudsley 3-item Depression Visual Analogue Scale (M3VAS).

**Fig. 2:**
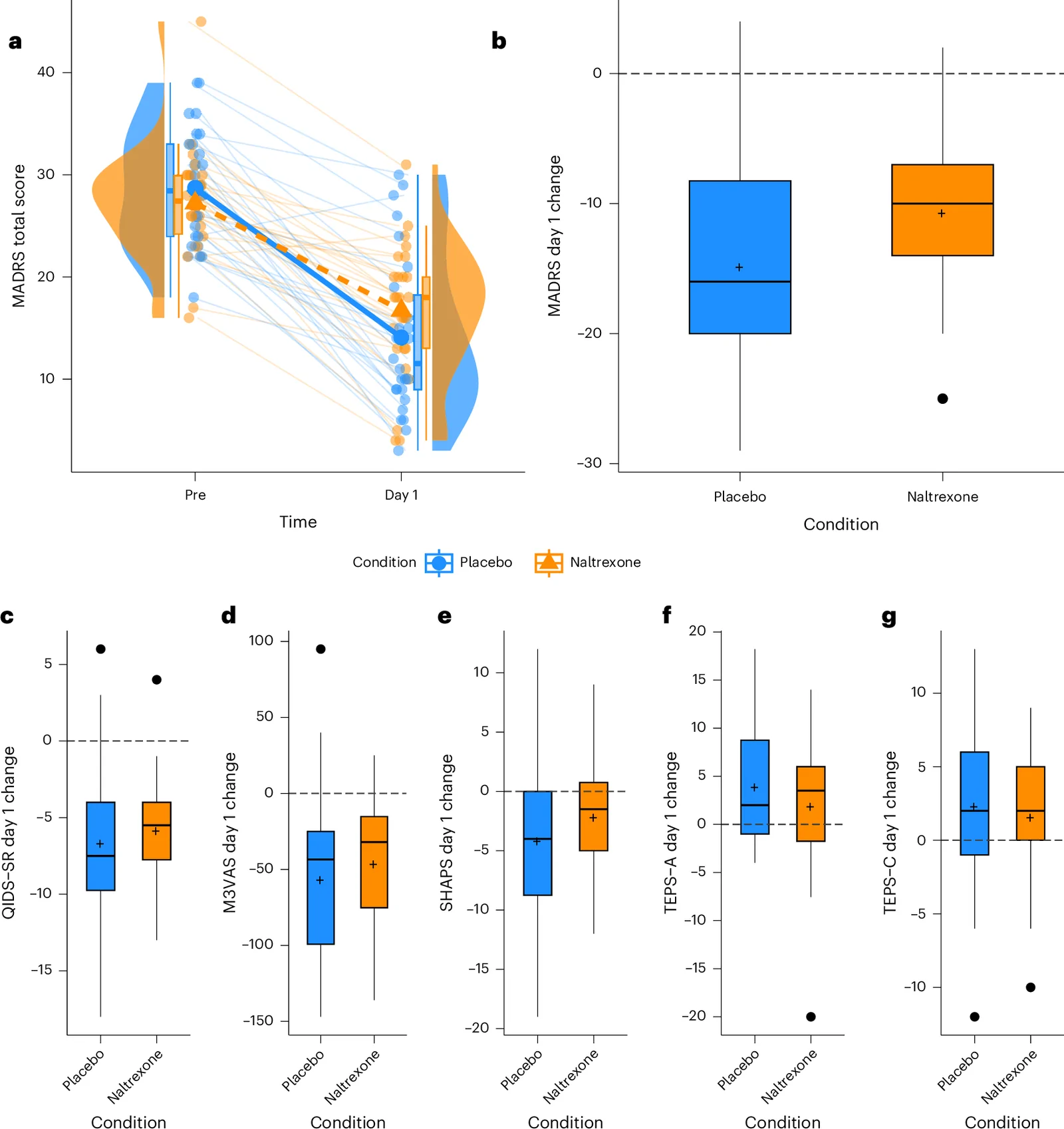
Clinical measure outcomes. **a**, MADRS total scores at preinfusion and at day 1 postinfusion. The thick lines represent the mean values for each condition, with individual data points connected by thinner lines. **b**, Change in MADRS scores from preinfusion to day 1 postinfusion. **c**, Change in QIDS–SR scores from preinfusion to day 1 postinfusion. **d**, Change in M3VAS scores from preinfusion to day 1 postinfusion **e**, Change in SHAPS scores from preinfusion to day 1 postinfusion. **f**, Change in TEPS-A scores from preinfusion to day 1 postinfusion. **g**, Change in TEPS-C scores from preinfusion to day 1 postinfusion. Note an increase in TEPS-A and TEPS-C scores indicates a reduction in anhedonia symptoms. Box plot elements: box spans the IQR (25th–75th percentile), central line is the median (50th percentile), + marks the mean, whiskers extend to the lowest and highest values within 1.5 × IQR of the box and values beyond that range are shown individually. Each plot shows data from *n* = 26 participants who completed the crossover.

**Extended Data Fig. 1:**
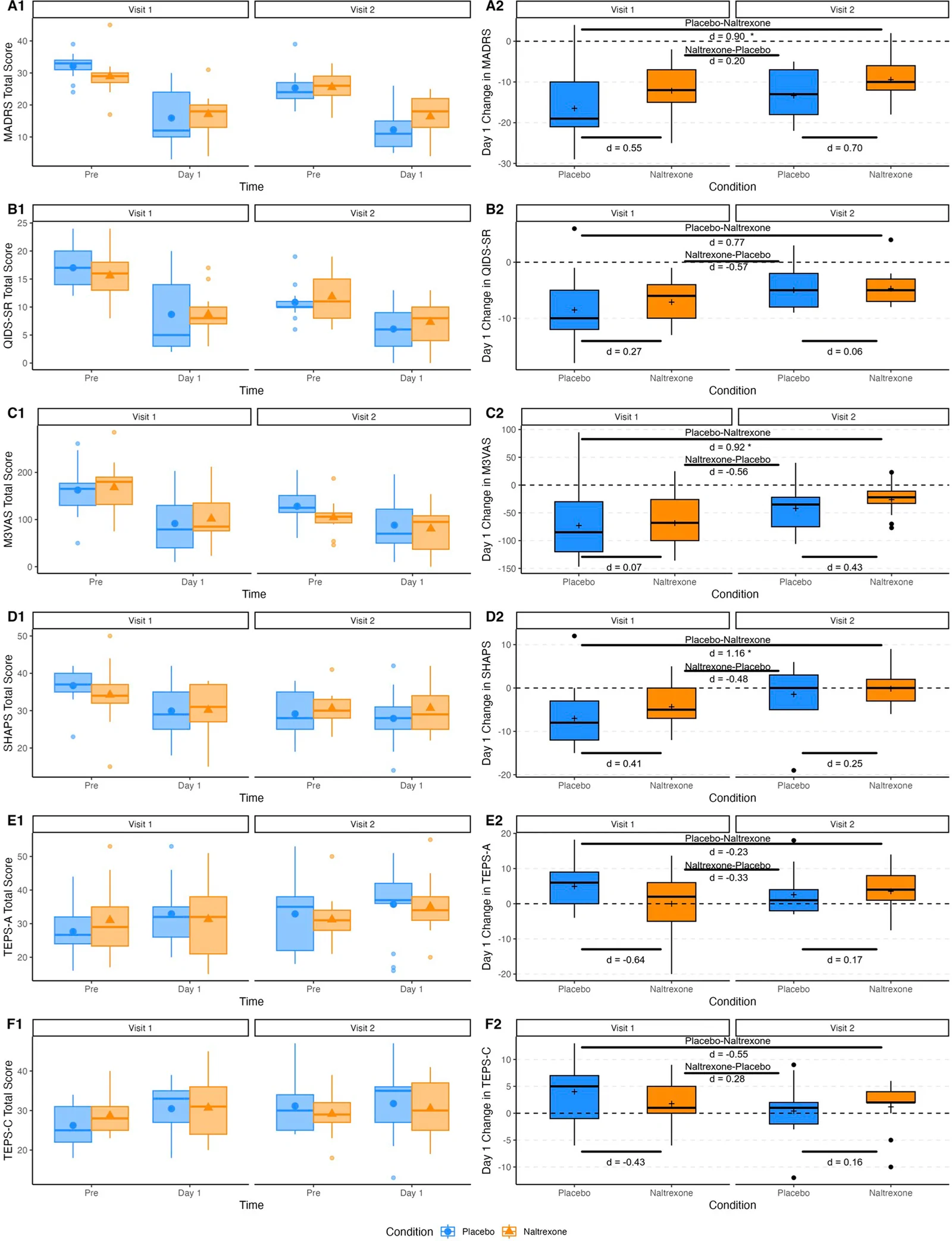
Clinical scores and change scores, by visit. MADRS, QIDS-SR, M3VAS, SHAPS, TEPS-A, and TEPS-C scores at pre-infusion and Day 1 post-infusion by visit (Panels A1—F1), and Day 1 change in scores by visit (Panels A2—F2). Effect sizes (Cohen’s *d*) and significance (*p < 0.05) are shown for Day 1 changes between placebo and naltrexone conditions, comparing Visit 1, Visit 2, Placebo-Naltrexone, and Naltrexone-Placebo orders. Independent t-tests were used for between-subject comparisons; paired t-tests for within-subject comparisons. Box plot elements: Box spans the interquartile range (IQR) (25th–75th percentile), central line is the median (50th percentile), ‘+’ marks the mean, whiskers extend to the lowest and highest values within 1.5 × IQR of the box, and values beyond that range are shown individually. Each plot shows data from n = 26 participants who completed the crossover.

There were no significant differences in mean baseline MADRS scores for the placebo-plus-ketamine condition (mean = 28.73, s.d. = 5.75) and the naltrexone-plus-ketamine condition (mean = 27.23, s.d. = 5.54). Linear mixed-effect model analysis revealed a significant main effect of time (*F*_1,74_ = 197.93, *P* < 0.001), with reductions in mean MADRS scores at day 1 postinfusion (placebo-plus-ketamine (mean = −14.65, s.d. = 7.77); naltrexone-plus-ketamine condition (mean = −10.50, s.d. = 5.91)) and a significantly attenuated reduction for the naltrexone-plus-ketamine condition (mean difference from placebo = 4.15, s.d. = 8.59, condition-by-time interaction, *F*_1,74_ = 5.39, *P* = 0.023; Cohen’s *d* = 0.60) (Fig. 2a,b). Finally, there was a significant effect of visit (*F*_1,74_ = 16.68, *P* = 0.001) with lower mean MADRS scores across the visit 2 period (predose and day 1 after the infusion) (mean = 19.87, s.d. = 8.06) compared to the visit 1 period (mean = 23.52, s.d. = 9.67) (Extended Data Table 1 and Extended Data Fig. 1).

For the self-report measures, there was a significant effect of time for the QIDS–SR (*F*_1,74_ = 96.43, *P* < 0.001) and for the M3VAS scores (*F*_1,74_ = 47.98, *P* < 0.001), with reductions in mean QIDS–SR scores (placebo-plus-ketamine (mean = −6.54, s.d. = 5.43); naltrexone-plus-ketamine condition (mean = −5.73, s.d. = 3.62)) and reductions in mean M3VAS scores at day 1 postinfusion (placebo-plus-ketamine (mean = −55.50, s.d. = 57.12); naltrexone-plus-ketamine condition (mean = −45.27, s.d. = 46.96)). There were no significant effects of condition (QIDS–SR: *F*_1,74_ = 0.11, *P* = 0.736; M3VAS: *F*_1,74_ = 0.28, *P* = 0.599) or condition-by-time interactions (QIDS–SR: *F*_1,74_ = 0.42, *P* = 0.520; M3VAS: *F*_1,74_ = 0.50, *P* = 0.484). The effect of visit was significant for the QIDS–SR (*F*_1,74_ = 31.05, *P* < 0.001) and M3VAS scores (*F*_1,74_ = 17.72, *P* < 0.001), with lower mean QIDS–SR scores across the visit 2 period (mean = 9.02, s.d. = 4.36) compared to the visit 1 period (mean = 12.50, s.d. = 6.20) and lower mean M3VAS scores across the visit 2 period (mean = 100.40, s.d. = 47.53) compared to the visit 1 period (mean = 131.02, s.d. = 64.40).

#### Anhedonia

Anhedonia was measured using the Snaith Hamilton Pleasure Scale (SHAPS) and the Temporal Experience of Pleasure Scale (TEPS), which provides separate anticipatory (TEPS-A) and consummatory (TEPS-C) subscale scores.

There was a significant effect of time for mean SHAPS scores (*F*_1,74_ = 12.87, *P* = 0.006) with reductions in mean SHAPS scores at day 1 postinfusion (placebo-plus-ketamine (mean = −4.00, s.d. = 7.47); naltrexone-plus-ketamine condition (mean = −2.00, s.d. = 4.96)) and no significant effect of condition (*F*_1,74_ = 0.36, *P* = 0.552) or condition-by-time interaction (*F*_1,74_ = 1.43, *P* = 0.236). There was a significant effect of visit for SHAPS scores (*F*_1,74_ = 14.22, *P* = 0.003), with lower mean SHAPS scores across the visit 2 period (mean = 29.60, s.d. = 6.11) compared to the visit 1 period (mean = 32.75, s.d. = 7.22).

There was a significant effect of time for TEPS-A (*F*_1,74_ = 8.84, *P* = 0.004) and TEPS-C scores (*F*_1,74_ = 7.89, *P* = 0.006), with increases in mean TEPS-A scores (placebo-plus-ketamine (mean = 4.07, s.d. = 6.52); naltrexone-plus-ketamine (mean = 2.08, s.d. = 7.41)) and increases in mean TEPS-C scores at day 1 postinfusion (placebo-plus-ketamine (mean = 2.42, s.d. = 5.73) and naltrexone-plus-ketamine condition (mean = 1.69, s.d. = 4.35)). There were no significant effects of condition (TEPS-A: *F*_1,74_ = 0.02, *P* = 0.881; TEPS-C: *F*_1,74_ = 0.03, *P* = 0.855) or condition-by-time interactions (TEPS-A: *F*_1,74_ = 0.93, *P* = 0.338; TEPS-C: *F*_1,74_ = 0.25, *P* = 0.619). There were significant effects of visit for both the TEPS-A (*F*_1,74_ = 8.47, *P* = 0.005) and TEPS-C scores (*F*_1,74_ = 4.98, *P* = 0.029), with higher mean TEPS-A scores across the visit 2 period (mean = 33.74, s.d. = 9.60) compared to the visit 1 period (mean = 30.73, s.d. = 10.18) and higher mean TEPS-C scores across the visit 2 period (mean = 30.65, s.d. = 6.95) compared to the visit 1 period (mean = 29.02, s.d. = 6.09).

#### Dissociation and psychotomimetic states

Subjective effects were measured using the Clinician Administered Dissociative States Scale (CADSS) and the self-report Psychotomimetic States Inventory (PSI). There were no significant differences in CADSS scores (*F*_1,25_ = 0.003, *P* = 0.959) for the placebo-plus-ketamine (mean = 31.62, s.d. = 13.23) and the naltrexone-plus-ketamine condition (mean = 31.77, s.d. = 12.62). Similarly, there were no significant differences in any PSI subscale scores between the placebo-plus-ketamine and naltrexone-plus-ketamine condition (Supplementary Fig. 2).

#### Evaluation of blind and adverse events

In the Placebo-Naltrexone order group, 54% (7 out of 13) of participants correctly guessed their pretreatment order, the same rate observed in the Naltrexone-Placebo group. The clinical assessor correctly identified the treatment order in 69% (9 out of 13) of cases in the Placebo-Naltrexone group and 54% (7 out of 13) in the Naltrexone-Placebo group. The James Blinding Index estimates were 0.46 (95% CI: 0.27–0.64) and 0.38 (95% CI: 0.20–0.57) for the participant and clinical assessor guesses, respectively. As the upper limits of these two-sided confidence intervals were both >0.5, blinding was considered successful (Supplementary Table 3). Aside from dissociation, the most common adverse events were nausea and vomiting along with headache and dizziness. There were no significant differences in adverse event data between the placebo-plus-ketamine and naltrexone-plus-ketamine sessions (Supplementary Table 4). There were no serious adverse events.

#### Exploratory analyses

Results from exploratory analyses are presented in the Supplementary Information: response and remission rates (MADRS and QIDS–SR) are in Supplementary Table 2, changes in self-report measures at days 3 and 7 postinfusion are in Supplementary Fig. 3 and subgroup analyses of depressive symptom reduction (MADRS and QIDS–SR) on day 1 postinfusion among participants who achieved a ≥50% reduction in the placebo-plus-ketamine condition are in Supplementary Fig. 4.

### ^1^H-fMRS results

**Fig. 3:**
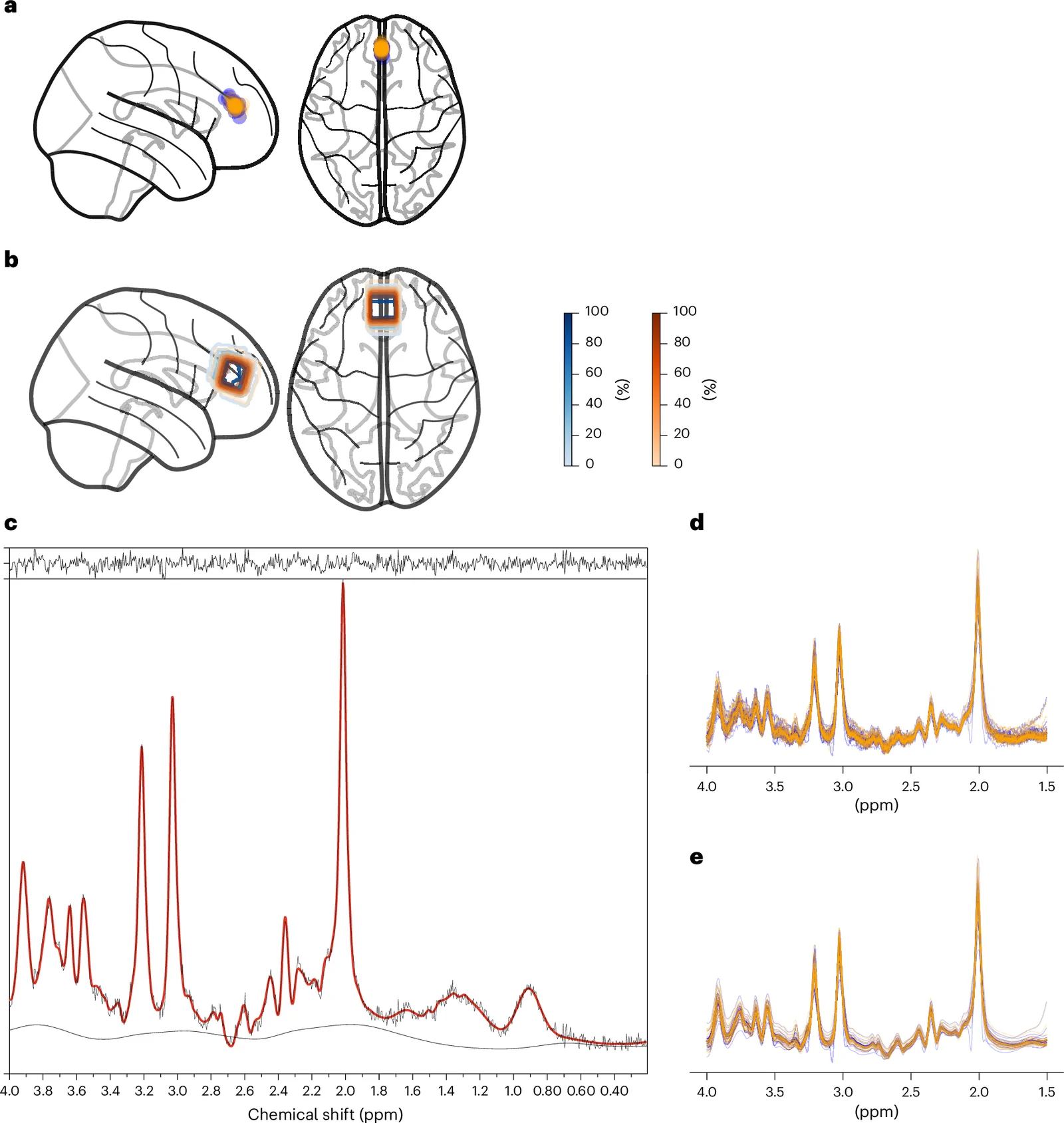
^1^H-fMRS voxel position and spectra. **a**, Voxel center placement. **b**, Voxel density map across participants and sessions in Montreal Neurological Institute (MNI) space. Blue, placebo condition; orange, naltrexone. The shading of the contour lines represents the percentage (%) overlap at that point. **c**, Sample spectrum from averaged block for single participant with output of the fit (red) overlaid on the acquired spectrum (black). The estimated baseline is displayed under the spectrum in black. Plotted in units of parts per million (ppm). **d**, Combined spectra for single block across participants and conditions. Blue, placebo; orange, naltrexone. **e**, Combined spectra fit for single block across participants and conditions. Blue, placebo; orange, naltrexone.

Twenty-four participants were included in the ^1^H-fMRS analyses after two participants were excluded due to missing data, spectral artifact or quality control failure. Our primary metabolite of interest was Glx, a combined measure of glutamate (Glu) and glutamine (Gln), expressed as a ratio to total *N*-acetylaspartate (tNAA: *N*-acetylaspartate plus *N*-acetylaspartylglutamate). We chose tNAA as the internal reference rather than the more common total creatine (tCr) because tNAA remained relatively stable across infusion blocks (Methods). The ¹H-fMRS voxel placement and representative spectra output are shown in Fig. 3.

**Fig. 4:**
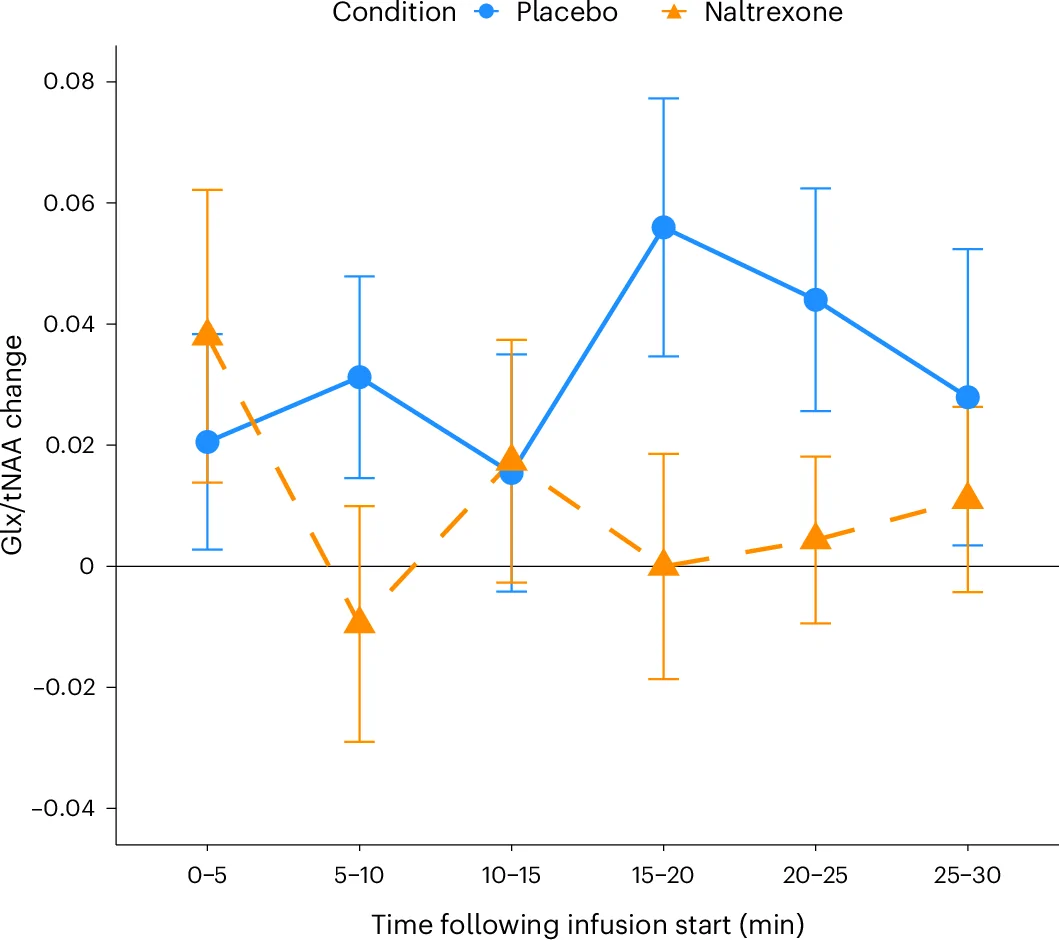
Mean Glx/tNAA change across ketamine-infusion blocks. Mean Glx/tNAA change in each of six infusion blocks relative to the preinfusion baseline block, shown separately for placebo and naltrexone pretreatment conditions. *n* = 24 participants with complete MRS data passing quality control. Error bars ± standard errors.

There were no significant differences in mean baseline Glx/tNAA for the placebo-plus-ketamine condition (mean = 1.34, s.d. = 0.11) and the naltrexone-plus-ketamine condition (mean = 1.33, s.d. = 0.12). For the Glx/tNAA change from preinfusion baseline, the linear mixed-effect model analysis revealed a significant main effect of condition (*F*_1,253_ = 4.83, *P* = 0.029; Cohen’s *d* = 0.34), with a higher mean increase in Glx/tNAA during the ketamine infusion for placebo compared to the naltrexone pretreatment condition (Fig. 4). There was no significant main effect of infusion block (*F*_5,253_ = 0.33, *P* = 0.896) or condition-by-block interaction (*F*_5,253_ = 1.29, *P* = 0.270).

### ^1^H-fMRS sensitivity analyses

**Fig. 5:**
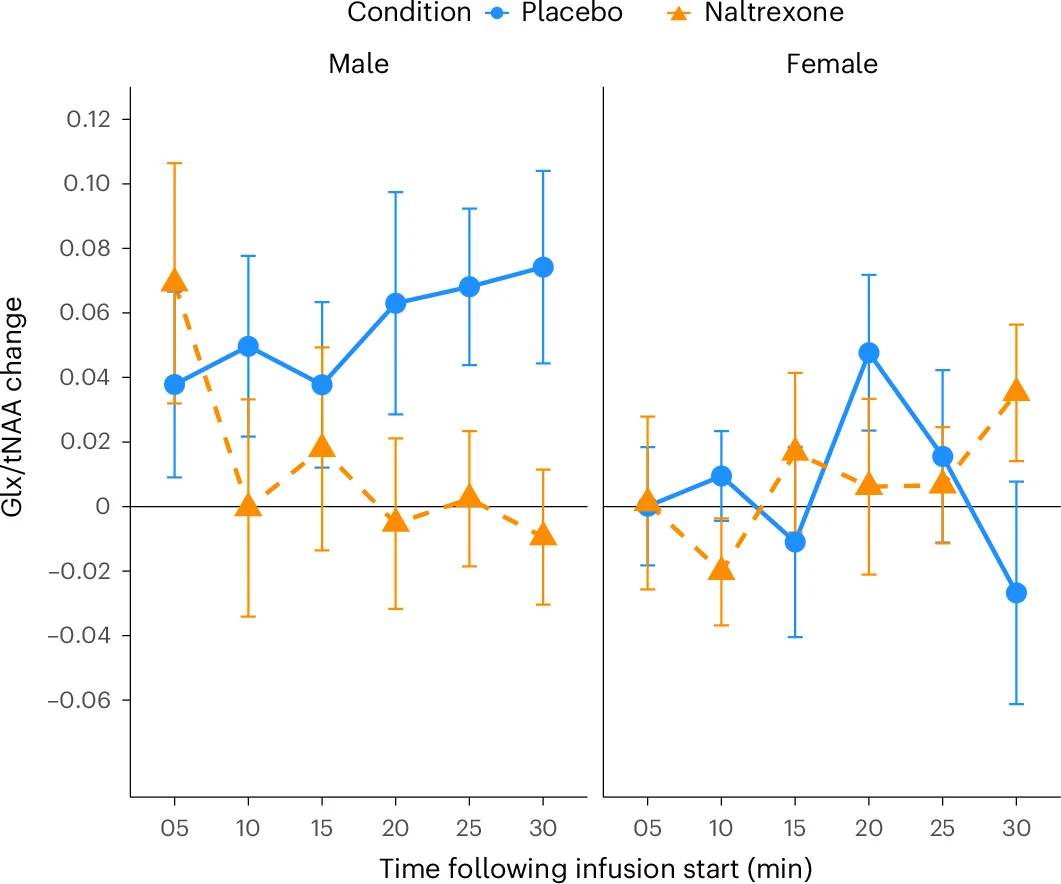
Mean Glx/tNAA change from baseline across ketamine-infusion blocks by sex. Mean Glx/tNAA change across six infusion blocks relative to the preinfusion baseline, shown separately for placebo and naltrexone pretreatment conditions, split by sex. Females, *n* = 11 and males, *n* = 13, with complete MRS data passing quality control. Error bars ± standard errors.

Separate post hoc linear mixed-effects models were conducted to assess the influence of potential confounders on metabolite measures. We evaluated the factors age, sex and gray-to-white matter composition within the MRS voxel, antidepressant status and pretreatment order, which might affect these measures^29–31^. In addition, MRS quality metrics revealed a significantly higher signal-to-noise ratio (SNR) in the naltrexone condition (mean = 37.15, s.d. = 4.92) compared to the placebo condition (mean = 35.83, s.d. = 6.12) (Supplementary Table 8). To address these factors, models were run individually, adjusting for (1) age, (2) sex, (3) gray-to-white matter fraction, (4) SNR, (5) antidepressant status and (6) pretreatment order. In each exploratory model, the main effect of condition remained significant (all *P* < 0.05). No significant effects were observed for age, gray-to-white matter fraction, SNR or antidepressant status. However, the model adjusted for sex revealed a significant condition-by-sex interaction on Glx/tNAA change (*F*_1,242_ = 4.81, *P* = 0.029), with differences between placebo and naltrexone pretreatment conditions being more pronounced in males than in females (Fig. 5). Furthermore, the model adjusted for pretreatment order revealed a significant condition-by-order interaction on Glx/tNAA change (*F*_1,242_ = 4.22, *P* = 0.041), with differences between placebo and naltrexone pretreatment conditions most pronounced in the Naltrexone-Placebo order group (Supplementary Fig. 9).

### Glx/tNAA and clinical measure correlations

Exploratory analyses revealed a positive relationship between Block-7 ΔGlx/tNAA and Peak ΔGlx/tNAA change and day 1 TEPS-A change scores for the placebo condition (*r* = 0.45, *P* = 0.026 and *r* = 0.51, *P* = 0.011) but not for the naltrexone condition (*r* = 0.05, *P* = 0.808 and *r* = −0.02, *P* = 0.932). However, neither of these correlations survived correction for multiple comparisons (Supplementary Fig. 12).

## Discussion

This study provides support for the hypothesis that ketamine administration leads to acute increases in glutamatergic activity in the ACC in individuals with depression and that pretreatment with the opioid receptor antagonist naltrexone attenuates this increase. Furthermore, the study provides additional clinical evidence that pretreatment with naltrexone may attenuate the antidepressant effects of ketamine measured one day after a single dose.

This study examined opioid antagonist pretreatment effects on brain glutamatergic activity after ketamine administration. Considering the placebo-plus-ketamine arm, our results support previous rodent studies suggesting ketamine increases frontal cortex glutamatergic activity^5–7^. Furthermore, these results are consistent with previous ^1^H-MRS studies in humans that found IV ketamine elevates ACC glutamine and Glx immediately^9,10^ and ACC glutamate levels after 35 min in healthy individuals^8^, together with studies in patients with MDD that reported an acute mPFC Glx increase^11^ and an increase prefrontal ^13^C-glutamine^13^ following IV ketamine administration. In the MDD studies, the ^13^C-glutamine enrichment increase was detected in the first 20 min (ref. ^13^) and Glx changes peaked above baseline in ~26 min (ref. ^11^), which aligns with our findings, where peak placebo-plus-ketamine Glx/tNAA changes were recorded in blocks 15–20 min and 20–25 min after the infusion start.

Our study demonstrated that naltrexone significantly attenuated the acute increase in Glx/tNAA during ketamine infusion compared to placebo, implying a potential interaction between opioidergic and glutamatergic systems, which may be of importance in understanding ketamine’s neurobiological effects. One hypothesis is that naltrexone may indirectly reduce ketamine-induced glutamate release by inhibiting MOR activation on GABAergic interneurons. Normally, MOR activation suppresses GABAergic signaling, leading to increased activity in glutamatergic pyramidal neurons^32–34^. However, naltrexone, as an opioid antagonist, may increase GABAergic interneuron activity, thereby enhancing GABAergic tone, which could lead to reduced glutamate release from pyramidal neurons and a consequent attenuation of ketamine’s acute excitatory effects. Recent preclinical work has found that ketamine acts as a potent allosteric modulator of opioid receptors, enhancing the effects of endogenous peptides at submicromolar levels^35^. Naltrexone’s disruption of this synergy may be another mechanism by which it reduces ketamine’s excitatory glutamate effects. It is important to note, since Glx/tNAA indexes the cortical glutamate + glutamine pool rather than synaptic glutamate release alone, the attenuation we observed may reflect metabolic changes as well as altered neurotransmitter cycling, consistent with ¹³C-MRS evidence that prefrontal tricarboxylic (TCA)-cycle activity can fall without a corresponding change in glutamate and glutamine cycling in depression^36^.

Alongside the attenuation of ketamine-induced glutamatergic activity, we found naltrexone pretreatment led to less marked reductions in clinician-rated MADRS scores at day 1 postinfusion (equating to a 28% attenuation in the main effect of ketamine). This finding aligns with work from Williams et al.^21^ that utilized a similar crossover design and demonstrated a significantly smaller reduction of mean 17-item Hamilton Depression Rating Scale (HAM-D) scores for a naltrexone-plus-ketamine compared to a placebo-plus-ketamine condition at day 1 postinfusion in individuals with treatment-resistant depression (*n* = 12) with a similar effect size (*d* = 0.7) to that found in this study (*d* = 0.6). In our study, although reductions in self-reported depressive measures (QIDS–SR and M3VAS) were numerically lower for the naltrexone-plus-ketamine condition compared to placebo at day 1 postinfusion, the differences were not statistically significant. The MADRS may be more sensitive to subtle changes in depressive symptoms than self-reported scales, which could explain the differing results^37^. Concerns about the QIDS–SR include potential imprecision due to compound items, reliance on unidimensional sum scoring and vague phrasing of scoring options that may result in greater variability^38^.

Taken together, our findings suggest an attenuation, but not a blocking, of ketamine’s acute antidepressant effects with naltrexone pretreatment in a sample of patients with MDD. We propose a potential indirect mechanism via which opioid antagonism may be reducing acute ketamine-induced glutamatergic activity. While a previous positron emission tomography (PET) study found that the magnitude of ketamine-induced glutamate release in the hippocampus predicts its antidepressant effects^39^, our findings, consistent with other MRS studies, did not reveal significant correlations between the glutamatergic changes and clinical response^11,40^. This may be due to the limited temporal window of our MRS measurements or could indicate that downstream mechanisms, such as AMPA receptor activation, neuroplastic signaling pathways (for example, BDNF or TrkB and ERK or mTOR) or effects in regions beyond the ACC, are more critical targets for mediating antidepressant response. Importantly, this study cannot rule out other mechanisms whereby naltrexone may modulate ketamine’s acute antidepressant response through alternative direct and/or indirect actions at the mu opioid receptors^41^.

The strengths of the study include its robust design and innovative methodology. The randomized, double-blind crossover design minimized variability by using each participant as their own control, allowing for more reliable comparisons of biological responses within individuals rather than between them. By employing ^1^H-fMRS, this enabled acute and dynamic changes of in vivo glutamatergic neurochemistry in response to ketamine and opioid receptor antagonism to be determined.

A limitation of this study is that the ^1^H-fMRS sequence did not include interleaved unsuppressed water acquisitions, which are necessary for reliable water-scaled metabolite quantification adjusted for tissue type or relaxation differences. Instead, tNAA was used as a reference for Glx values in each block to mitigate potential confounds, such as scanner drift, which would not be adequately corrected using only the water unsuppressed acquisitions from the end of the scan. tNAA and tCr are frequently chosen as internal references as they are assumed to remain relatively stable between experimental conditions and over time^42^. However, this assumption warrants caution, especially for tCr, as creatine is involved in bioenergetics and levels may be influenced by activity and vascularization^43^. In this study, we found tCr increased across ketamine-infusion blocks while tNAA remained relatively stable. Changes in creatine have also been reported following amphetamine administration and electroconvulsive therapy^44,45^, therefore ketamine’s potential effects on creatine and phosphocreatine cycling warrant further study. In adition, this ^1^H-fMRS study was performed at 3 Tesla where glutamate and glutamine may not be reliably differentiated, thus highlighting the need for future ^1^H-fMRS ketamine studies to be conducted at higher magnetic field strengths.

Although our analyses showed no significant differences in baseline Glx/tNAA (or Glu/tNAA or Gln/tNAA) between the naltrexone and placebo groups measured 5 min before the ketamine infusion, suggesting that naltrexone does not independently affect glutamatergic measures at that early time point, our study was not designed with a dedicated placebo-infusion arm to assess its effects over the duration of the infusion. Future studies should include such a control group to determine naltrexone’s independent impact on cortical glutamate measures over time and to clarify any interactions with ketamine.

In our exploratory analyses, we observed a significant condition-by-sex interaction for Glx/tNAA change, with male participants showing more pronounced differences between placebo and naltrexone pretreatment conditions compared to females. Despite limited investigation of sex and other biological variables potentially influencing ketamine’s opioid-dependent therapeutic efficacy, preliminary preclinical work suggests subanesthetic ketamine evokes opioid-mediated behavioral and neurophysiological effects in male but not female rats^46^. Our study sample was not powered to fully evaluate each sex separately; however, these initial results highlight the importance of focusing on sex as a biological variable in future studies on the clinical and neurobiological effects of ketamine.

Limitations in study design and sample characteristics may partly explain why naltrexone did not significantly alter the effects of ketamine on anhedonia or self-reported depression measures. In our study, the average interval between visits was 19 days, compared to 33 days in Williams et al.^21^. We observed significant session effects, with lower preinfusion symptom scores at the second visit, suggesting that symptoms had not fully returned to baseline. This attenuation of baseline symptoms may have reduced the impact of ketamine’s effects during the second visit, potentially masking differences between the naltrexone and placebo conditions. Supplementary analyses (Extended Data Fig. 1) showed that naltrexone attenuated ketamine’s effects on most clinical measures during visit 1. Across both visits, this attenuation was particularly evident in participants in the ‘Placebo-Naltrexone’ treatment order group. In contrast, in participants who received naltrexone first, the effect of naltrexone appeared diminished or even reversed, although this was not statistically significant. Together, these findings suggest that extending the interval between visits to allow symptoms to return fully to baseline could have improved study power to clarify potential differences in self-reported depression and anhedonia scores between the naltrexone and placebo conditions. Finally, our sample had a lower mean number of failed antidepressant treatments compared to Williams et al.^21^, potentially indicating less treatment resistance. The possibility that varying levels of treatment resistance may influence responsiveness to opioid-mediated treatments warrants further investigation.

Our study used racemic ketamine so it was not possible to distinguish the individual contributions of (*S*)-ketamine and (*R*)-ketamine. (*S*)-ketamine exhibits higher affinity and potency at MORs than (*R*)-ketamine, and each enantiomers and their metabolites may exert antidepressant effects via distinct mechanisms^15^. Preclinical work has demonstrated the abuse liability of (*R*,*S*)-ketamine is mediated primarily by (*S*)-ketamine and involves direct interactions with MORs at doses and concentrations relevant to its reinforcement and antidepressant-like efficacy^18^. In addition, our study did not include direct pharmacokinetic measurements to assess interactions between ketamine and naltrexone. Ketamine is primarily metabolized by the liver enzymes CYP2B6 and CYP3A4 (ref. ^16^) whereas naltrexone is mainly processed by dihydrodiol dehydrogenases to produce its active metabolite, 6-β-naltrexol^47^. As these drugs are metabolized by different enzymatic systems, a substantial pharmacokinetic interaction is unlikely. Future clinical research should investigate the effects of opioid antagonism on the individual ketamine enantiomers and include measurements of relevant metabolites to confirm that observed effects are due primarily to pharmacodynamic interactions rather than alterations in the metabolism or clearance of ketamine.

Finally, the mu opioid system is known to influence the placebo response in antidepressant treatments^48,49^, and previous research has shown that naltrexone can partially reduce this response^50^. However, our study did not include a placebo-infusion arm, leaving it unclear whether naltrexone would have affected the glutamatergic or antidepressant effects of placebo infusions. In addition to retrospective blinding assessments, it would have been beneficial to evaluate blinding after each study phase and consider participant expectations before each infusion. This approach could have helped explore potential interactions between expectancy and opioid system modulation.

## Conclusion

The possibility of an opioid mechanism underlying the antidepressant mechanisms of ketamine has been a recent subject of considerable debate. Our study provides evidence that opioid system activation may contribute to the acute antidepressant effects of ketamine. Furthermore, we elucidate a possible mechanism through which naltrexone may attenuate the antidepressant effects of ketamine by demonstrating opioid modulation of ketamine-induced glutamatergic activity. Future work should examine interactions between the glutamatergic and opioidergic systems to better understand the potential synergistic or independent contributions of these systems to ketamine response.

## Methods

### Study design and procedures

Ethical approval was obtained from the London – City & East Research Ethics Committee (Reference: 21/LO/0334), and the study was jointly sponsored by King’s College London and South London and Maudsley NHS Foundation Trust. After receiving a complete description of the study, all participants provided informed written consent (ClinicalTrials.gov registration: NCT04977674).

This was a double-blind, randomized, placebo-controlled, crossover study in which each participant completed two treatment sessions. In one session, participants received an oral placebo (ascorbic acid 50 mg) before a ketamine infusion (0.5 mg per kg administered over 40 min) during magnetic resonance imaging (MRI); in the other session, they received oral naltrexone (50 mg) before the same ketamine-infusion protocol. A washout interval of 2 to 4 weeks was maintained between each arm of the crossover to minimize potential carryover effects. Participants were assigned to one of two treatment orders—either placebo in the first session followed by naltrexone in the second or vice versa—using an online randomization system provided by the King’s Clinical Trials Unit. The random sequence was generated using block randomization with a fixed block size of four and stratification by sex to ensure balanced treatment order distribution. Pharmacy staff, who had access to unblinded treatment assignments, over-encapsulated both the placebo and naltrexone pills to ensure identical appearance and maintain blinding for both participants and investigators. Unblinding only occurred once all participants had completed both arms of the crossover and data collection was complete.

Placebo or naltrexone was administered 1 hour before starting the ketamine infusion to ensure peak naltrexone levels at the initiation of the ketamine infusion^51^. The ketamine infusion was administered intravenously over 40 min, while participants were lying supine in the MRI scanner. Participants were monitored continuously with a respiration transducer, pulse oximetry and observed by a study physician throughout the scanning session.

### Participants

Participants between the ages of 18 and 50 were eligible if they met Diagnostic and Statistical Manual of Mental Disorders (fifth edition) criteria for a current single or recurrent episode of MDD, without psychotic features, based on clinical assessment and as documented by the Mini-International Neuropsychiatric Interview (v.7.0.2)^52^. Recruitment was conducted through referrals from primary care and secondary psychiatry services, online advertisements and word of mouth. Participants were all outpatients and for initial enrollment were required to have a score ≥18 on the 17-item HAM-D and have a history of inadequate response to two or more antidepressants, specifically listed in Section A: Recognized Antidepressants of the Maudsley Treatment Inventory^53^, prescribed at a minimum effective dose for at least 6 weeks, or at least one antidepressant prescribed at the minimum effective dose for at least 6 weeks and a course of evidence-based psychotherapy. To be eligible, participants were required to taper off any drugs likely to interact with glutamate or opioid system at least 14 days before starting the study (antipsychotics, anticonvulsants, mood stabilizers, gabapentinoids, opiates, opioid agonists/antagonists/combinations and stimulants). One exception was any regular antidepressant therapy the participant may be taking, providing this was stable for ≥4 weeks before screening (apart from monoamine oxidase inhibitors (MAOIs) that were not permitted). Other inclusion criteria included English literacy, right-handedness, body mass index of 18–30 kg per m^2^, weight 50–100 kg and the ability to tolerate MRI scanning procedures.

Exclusion criteria included: pregnancy or breastfeeding; history of schizophrenia, schizoaffective or bipolar disorder; current drug or alcohol dependence; a positive urine drug screen (for substances including ketamine, opiates, methadone, cocaine, amphetamines, benzodiazepines or cannabinoids) or a positive breath alcohol test at the screening or dosing visits; history of suicide attempt in the past year, or high risk of suicidal behavior on the Columbia-Suicide Severity Rating Scale^54^; history of nonresponse or intolerance to ketamine; clinically relevant electrocardiographic, urinalysis or biochemical abnormalities, including thyroid dysfunction; hypertension; history of cardiovascular, cerebrovascular or any other uncontrolled physical illness (including chronic pain conditions); self-reported neurological disorder; head injury resulting in loss of consciousness or any contraindication to MRI. Finally, use of compounds that may be affected by ketamine (diazepam, warfarin, carbamazepine, phenytoin, theophylline and levothyroxine) was not permitted.

Participants were required to abstain from alcohol from 24 hours before until 24 hours after each dosing, refrain from caffeine and nicotine on imaging visit days and not drive or operate machinery from the time of dosing until 24 hours postdose. Participants received £150 in compensation to acknowledge their time and effort in this research.

### Clinical and subjective measures

Clinician and self-reported measures of depressive symptoms and anhedonia were measured 2 hours before each ketamine infusion and at day 1 postinfusion, as previous studies have found antidepressant effects typically peak around 24 hours after a single dose of ketamine^55^. Rating scales included the 10-item MADRS^56^, a clinician‐rated measure of depressive severity; the 16-item QIDS–SR^57^, a self-report instrument covering depressive symptoms; the M3VAS^58^, a self-rated scale to measure mood, anhedonia and suicidality; the SHAPS, a 14-item self-rated scale used to measure the state of anhedonia^59^, with higher scores indicating higher levels of anhedonia and the TEPS, an 18-item self-rated scale used to assess anticipatory (TEPS-A) and consummatory components (TEPS-C) of pleasure^60^, with lower scores indicating higher levels of anhedonia. MADRS response was defined as a reduction from preinfusion score of ≥50% and remission was defined as a MADRS score ≤10. QIDS–SR response was defined as a reduction from preinfusion score of ≥50% and remission was defined as a QIDS–SR score ≤5. Alongside day 1 postinfusion, self-report measures (QIDS–SR, M3VAS, SHAPS and TEPS) were collected at days 3 and day 7 postinfusion as exploratory outcomes.

Subjective effects were measured after the ketamine infusion and MRI scan was complete, with participants asked to recall peak drug effects. These scales included the 23-item Clinician Administered Dissociative States Scale (CADSS)^61^ and the self-report PSI^62^.

Blinding was assessed by asking participants which order of placebo and naltrexone pretreatments they believed they received after the final dosing session at the day 1 follow-up, with ‘Don’t know’ a valid option. The clinical assessor similarly recorded their guess at this time point.

### ^1^H-fMRS data acquisition

The data were collected at 3 Tesla on a General Electric Discovery MR750 magnetic resonance scanner equipped with a 32-channel receive-only head coil at the NIHR King’s Clinical Research Facility, UK. A high-resolution sagittal T1-weighted (T1-w) 3D sagittal inversion recovery prepared spoiled gradient echo (IR-SPGR) scan was initially acquired for localization of the spectroscopy voxel (TR = 7.35 ms, TE = 3.04 ms, inversion time (TI) = 400 ms, field of view (FOV) = 270 mm, flip-angle = 11°, matrix size = 256 × 256, slice thickness = 1.2 mm, 196 slices). The ^1^H-fMRS voxel was positioned in an ACC region-of-interest (20 mm × 20 mm × 20 mm) with the center of the voxel placed 16 mm above the most anterior portion of the genus of the corpus callosum, perpendicular to the anterior commissure–posterior commissure line to minimize inclusion of white matter and cerebral spinal fluid (Supplementary Fig. 6). The positioning of this ACC voxel followed the same procedure and used the same orientation as in our previous ^1^H-MRS ketamine study^8^.

Auto-prescan was performed twice before each ^1^H-fMRS scan for optimization of water suppression and shimming. ^1^H-fMRS spectra were acquired continuously for a 5-min baseline period and during the initial 30 min of the ketamine infusion using point resolved spectroscopy, with chemical selective suppression for water suppression and outer volume suppression with very selective suppression pulses (TR = 2,000 ms, TE = 40 ms, 8-step phase cycle, 1,040 transients out of 16 water unsuppressed transients) (Fig. 1). Further details are provided in the minimum reporting standards for in vivo magnetic resonance spectroscopy checklist (Supplementary Table 7), according to consensus recommendations^63^.

### Anatomical registration

To assess the consistency of MRS voxel tissue composition, the voxels were coregistered to the T1-weighted image and segmented using the Gannet CoRegStandAlone function^64^, which calls SPM12 (ref. ^65^) to estimate the voxel fraction of gray matter, white matter and cerebrospinal fluid for each condition. The MRS voxels were spatially normalized to the MNI152 template image using the SPM12 spatial normalization function. This process involved applying the deformation fields derived from the T1-weighted images to which each MRS voxel had been coregistered. Subsequently, each voxel was converted to a binary mask using FSL. These binary masks were then combined to illustrate the positional overlap among participants and across conditions^66^ (Fig. 3a,b and Supplementary Table 9).

### ^1^H-fMRS processing

Data were preprocessed using an automated Free Induction Decay Appliance (FID-A) pipeline, which includes coil combination, removal of motion corrupted scans and spectral registration for frequency and phase drift correction^67,68^. ^1^H-fMRS data were averaged per subject into seven blocks (a baseline block and six blocks during the ketamine infusion), each lasting 288 s (144 transients (18 × 8-step phase cycle)). If motion corrupted scans were identified, the relevant transients were removed before determining the affected block average, ensuring the start time of each block remained consistent across participants and conditions. Averaged spectra were analyzed using LCModel v.6.3–1N^69^. The basis set was simulated on an 81^3^ grid using the fast 1-D spatial projection method^70^, using the FID-A toolbox and based on sequence timing and radiofrequency (RF) pulse shapes and included: alanine, ascorbic acid, aspartate, creatine, phosphocreatine, GABA, glucose, glutamine, glutamate, glutathione, glycerophosphocholine, myo-inositol, l-lactate, *N*-acetylaspartate, *N*-acetylaspartylglutamate, phosphocholine, phosphoethanoamine, scyllo-inositol and taurine with the default macromolecule stimulation parameters from LCModel.

The primary metabolite of interest was Glx (a combined measure of Glu and Gln) expressed relative to tNAA, the internal reference. tNAA was used as an internal reference to minimize the influence of changes during data acquisition (for example, scanner drift, change in linewidth and chemical shift displacement)^42^, which is beneficial to a within-subject design. In our study registration (ClinicalTrials.gov registration: NCT04977674) we planned to use tCr (creatine + phosphocreatine) as the internal reference; however, on examining changes in tCr and tNAA values (with respect to the water reference), it was found tCr increased across infusion blocks while tNAA remained relatively stable (Supplementary Fig. 7). Quality control procedures involved visual inspection of spectra alongside assessment of SNR, spectral linewidth (full width at half-maximum) and metabolite Cramér-Rao lower bounds. All averaged spectra included in the analyses had full-width at half-maximum ≤ 0.1 ppm (ref. ^71^), SNR ≥ 20 and Cramér-Rao lower bounds for Glx ≤ 20%.

Changes in Glx/tNAA for each infusion block, compared to the baseline block, were determined. The analysis focused on Glx/tNAA, as at 3 Tesla field strength Glu and Gln may not be reliably differentiated. However, for completeness, results for Glu/tNAA and Gln/tNAA are presented in the Supplementary Information (Supplementary Fig. 10).

### Statistical methods

A linear mixed-effects model for repeated measures was used for the primary outcome, Glx/tNAA change from baseline. The following fixed effects were included in the model: condition (placebo and naltrexone), block (six ketamine-infusion blocks) and the interaction between condition and block. Participant identification was included as a random intercept in this and subsequent models. For the day 1 clinical measure scores (MADRS, QIDS–SR, M3VAS, SHAPS, TEPS-A and TEPS-C), linear mixed-effects models were also used with condition (placebo and naltrexone), time (preinfusion and day 1 postinfusion), visit (visit 1 and visit 2) and the interaction between condition and time as fixed effects. For the TEPS-A subscale, the final subitem question was missing for the first four participants at the predose time point. We used within-person mean imputation to estimate these missing values, based on each participant’s mean response to the other TEPS-A items, before calculating the total TEPS-A score. For the subjective measure scores (CADSS and PSI), as these were only measured at one time point for each condition, only condition was included as a fixed effect. Effect sizes were calculated using standardized mean differences between conditions for the primary outcome (that is, Glx/tNAA change from baseline) and any relevant changes in day 1 clinical measure scores. The James Blinding Index was determined for participant and assessor pretreatment order guesses, where unblinding may be claimed if the upper limit of the index estimate’s two-sided confidence interval is <0.5 (ref. ^72^). All analyses were performed using R software (v.4.2.1) and the nlme package^73^ was used for linear mixed-effects modeling. An alpha of 0.05 (two-tailed) was used to determine statistical significance.

Exploratory linear mixed-effects analyses were conducted to examine changes in Glu/tNAA and Gln/tNAA from baseline across infusion blocks, using the same model parameters as the primary Glx/tNAA change analysis. Additionally, further analyses investigated potential relationships between changes in Glx/tNAA and clinical measure scores. These analyses focused on changes in Glx/tNAA from baseline across six infusion blocks (Block 2 to Block 7), and the mean and peak changes in Glx/tNAA. The changes were correlated with day 1 changes in clinical and anhedonia scales (MADRS, QIDS–SR, M3VAS, SHAPS, TEPS-A and TEPS-C). Pearson’s correlation coefficients were used for placebo and naltrexone conditions. A Bonferroni correction was applied for multiple comparisons, setting a significance threshold at *P* < 0.00625 (0.05 divided by 8). Additional exploratory linear mixed-effects models assessed changes in self-report measures (QIDS–SR, M3VAS, SHAPS, TEPS-A abd TEPS-C) at days 3 and day 7 postinfusion and between pretreatment conditions. Final analyses examined the reduction of depressive symptoms (MADRS and QIDS–SR) on postinfusion day 1 among participants who met the response criterion (≥50% reduction from preinfusion score) during the placebo-plus-ketamine condition, mirroring the primary outcome analysis by Williams et al.^21^.

### Reporting summary

Further information on research design is available in the Nature Portfolio Reporting Summary linked to this article.

## Online content

Any methods, additional references, Nature Portfolio reporting summaries, source data, extended data, supplementary information, acknowledgements, peer review information; details of author contributions and competing interests; and statements of data and code availability are available at https://doi.org/10.1038/s41591-025-03800-w.

## Supplementary information

Supplementary Information Supplementary Figs. 1–12 and Tables 1–9.

Reporting Summary

## Figures and Tables

**Extended Data Table 1:**
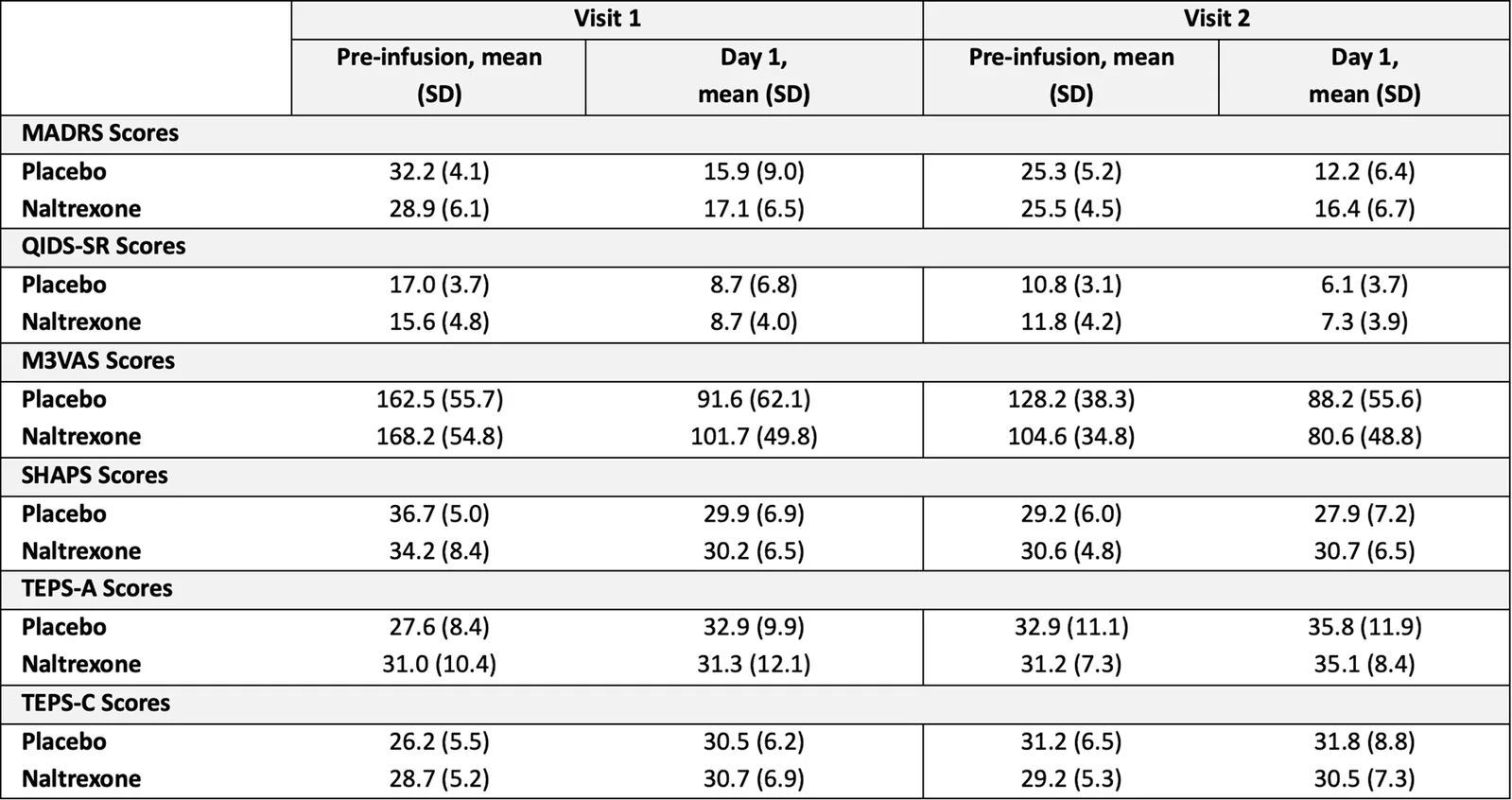
Mean clinical scores by visit

MADRS, QIDS-SR, M3VAS, SHAPS, TEPS-A, and TEPS-C scores at pre-infusion and Day 1 post-infusion by visit. Values are presented as mean ± standard deviation (SD).

## Data Availability

De-identified participant data is accessible via the Open Science Framework at https://osf.io/96gxt/. All participants have provided consent for their de-identified data to be shared with external entities for scientific research purposes.

## Code availability

R code used for data analysis is available via the Open Science Framework at https://osf.io/96gxt/.
