# Supplementary material for: Effect of naltrexone pretreatment on ketamine-induced glutamatergic activity and symptoms of depression: a randomized crossover study
Source: Nat Med. 2025 Jul 24;31(9):2958–66. doi: 10.1038/s41591-025-03800-w (PMC12443602; doi:10.1038/s41591-025-03800-w)
Supplement: Supplementary file 1 — Supplementary Figs. 1–12 and Tables 1–9. [file 41591_2025_3800_MOESM1_ESM.pdf]

nature medicine

Supplementary information

https://doi.org/10.1038/s41591-025-03800-w

Effect of naltrexone pretreatment on ketamine-induced glutamatergic activity and symptoms of depression: a randomized crossover study

In the format provided by the authors and unedited

Study flowchart

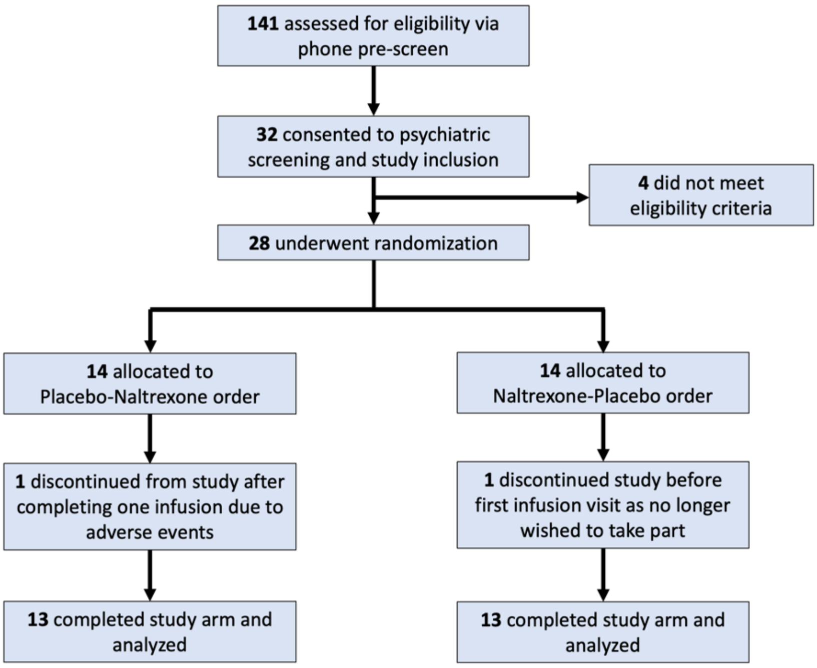

Figure S1: Study flowchart of recruitment and participation

Psychotropic medications

Table S1: Regular psychotropic medication received during the study

**Psychotropic medication**
Sertraline, n = 3
Venlafaxine, n = 3
Fluoxetine, n = 1
Citalopram, n = 1
Escitalopram, n = 1
Duloxetine, n = 1
Mirtazapine, n = 1
Agomelatine, n = 1

Response and remission rates

Table S2: Response and remission rates

	Day 1			Day 3			Day 7		
	n (%) placebo	n (%) naltrexone	p-value	n (%) placebo	n (%) naltrexone	p-value	n (%) placebo	n (%) naltrexone	p-value
**MADRS**									
Response	14 (54)	7 (27)	0.089	---	---	---	---	---	---
Remission	12 (46)	4 (15)	0.034	---	---	---	---	---	---
**QIDS-SR**									
Response	13 (50)	9 (35)	0.400	14 (54)	10 (38)	0.404	10 (38)	9 (35)	1.000
Remission	13 (50)	8 (31)	0.258	12 (46)	8 (31)	0.393	7 (27)	6 (23)	1.000

MADRS response was defined as reduction from pre-infusion score of ≥50% and remission defined as MADRS score ≤10. QIDS-SR response was defined as a reduction from pre-infusion score of ≥50% and remission defined as QIDS-SR score ≤5. Proportions of response/remission were compared using Fisher's exact test.

CADSS and PSI Scores

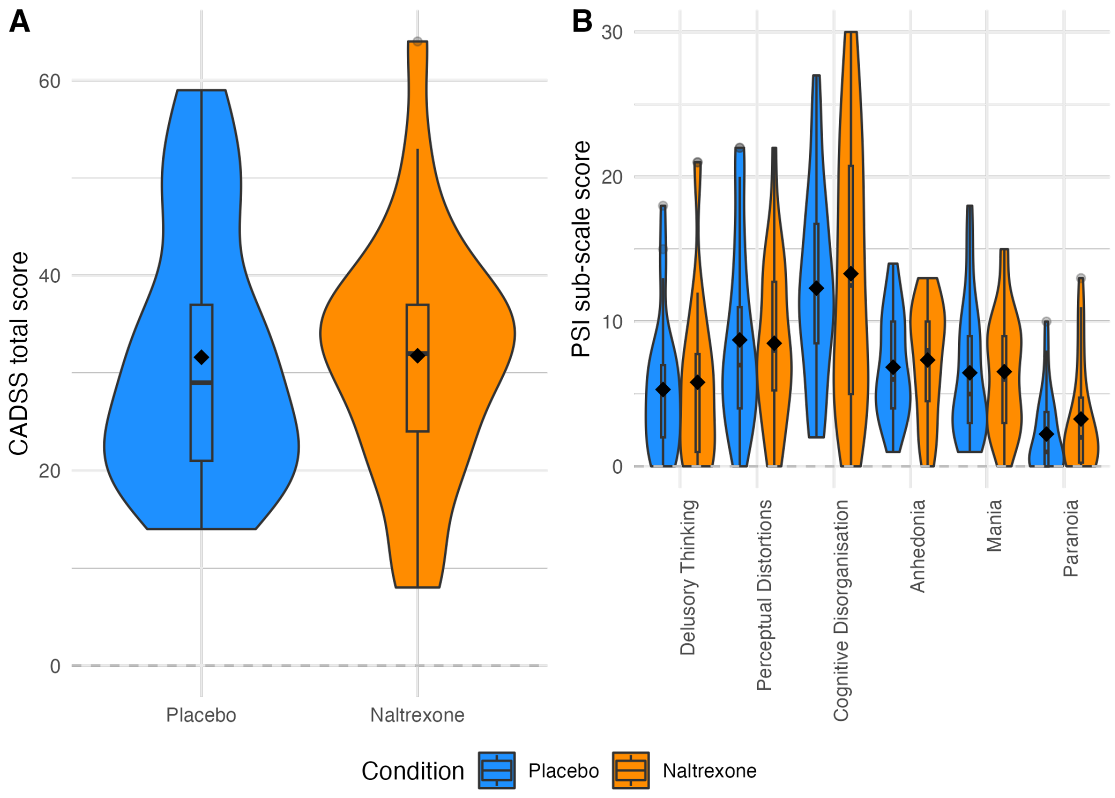

Figure S2: A) Clinician-Administered Dissociative States Scale (CADSS) scores B) Psychotomimetic States Inventory (PSI) subs-scale scores. Each plot shows data from n = 26 participants who completed the crossover.

Blinding

Table S3: Blinding assessment

**Participant**			
**Order guess**	**Placebo-Naltrexone group (n=13)**	**Naltrexone-Placebo group (n=13)**	**BI_{James}**
Placebo-Naltrexone - n (%)	7 (54)	6 (46)	
Naltrexone-Placebo - n (%)	3 (23)	7 (54)	
Don't know - n (%)	3 (23)	0 (0)	
Correct -n (%)	7 (54)	7 (54)	*Estimate:* 0.46 95% CI (0.27-0.64)
**Assessor**			
**Order guess**	**Placebo-Naltrexone group (n=13)**	**Naltrexone-Placebo group (n=13)**	**BI_{James}**
Placebo-Naltrexone - n (%)	9 (69)	6 (46)	
Naltrexone-Placebo - n (%)	4 (31)	7 (54)	
Don't know - n (%)	0 (0)	0 (0)	
Correct - n (%)	9 (69)	7 (54)	*Estimate*: 0.38 95% CI (0.20-0.57)

BI_{James} = James Blinding Index, where unblinding may be claimed if the upper limit of the two-sided confidence interval is <0.5

Adverse events

Table S4: Adverse event data

	Naltrexone	Placebo	p	test
n	26	27		
Dissociation — no. (%)	20 (76.9)	20 (74.1)	1.000	
Headache — no. (%)	5 (19.2)	7 (25.9)	0.800	
Dizziness — no. (%)	4 (15.4)	5 (18.5)	1.000	
Nausea — no. (%)	13 (50.0)	6 (22.2)	0.069	
Vomiting — no. (%)	3 (11.5)	2 (7.4)	0.669	exact
Lethargy — no. (%)	3 (11.5)	0 (0.0)	0.111	exact
Covid infection — no. (%)	0 (0.0)	1 (3.7)	1.000	exact
Hypoglycaemia — no. (%)	0 (0.0)	1 (3.7)	1.000	exact
Flu-like symptoms — no. (%)	1 (3.8)	0 (0.0)	0.491	exact
Urinary incontinence — no. (%)	0 (0.0)	1 (3.7)	1.000	exact

Self-report clinical measure scores post-infusion days 1, 3 and 7

Exploratory mixed-effects models including day 3 and day 7 self-report scores showed significant time and visit effects ($p < 0.05$) for all measures, with no significant condition or condition-by-time interactions. (**Table S5 and Figure S3**).

Table S5: Linear Mixed Model Results for Self-report Measure Scores

		numDF	denDF	F-value	p-value
**QIDS-SR Scores**	**Time**	3	174	53.70	<0.001
	Condition	1	174	0.44	0.510
	**Visit**	1	174	41.94	<0.001
	Condition x Time	3	174	0.19	0.905
**M3VAS Scores**	**Time**	3	174	24.02	<0.001
	Condition	1	174	0.03	0.876
	**Visit**	1	174	26.87	<0.001
	Condition x Time	3	174	0.33	0.803
**SHAPS Scores**	**Time**	3	174	8.18	<0.001
	Condition	1	174	1.71	0.193
	**Visit**	1	174	10.88	0.001
	Condition x Time	3	174	1.19	0.315
**TEPS-A Scores**	**Time**	3	174	5.82	<0.001
	Condition	1	174	0.99	0.320
	**Visit**	1	174	18.83	<0.001
	Condition x Time	3	174	0.91	0.440
**TEPS-C Scores**	**Time**	3	174	5.20	0.002
	Condition	1	174	1.47	0.226
	**Visit**	1	174	8.02	0.005
	Condition x Time	3	174	1.02	0.385

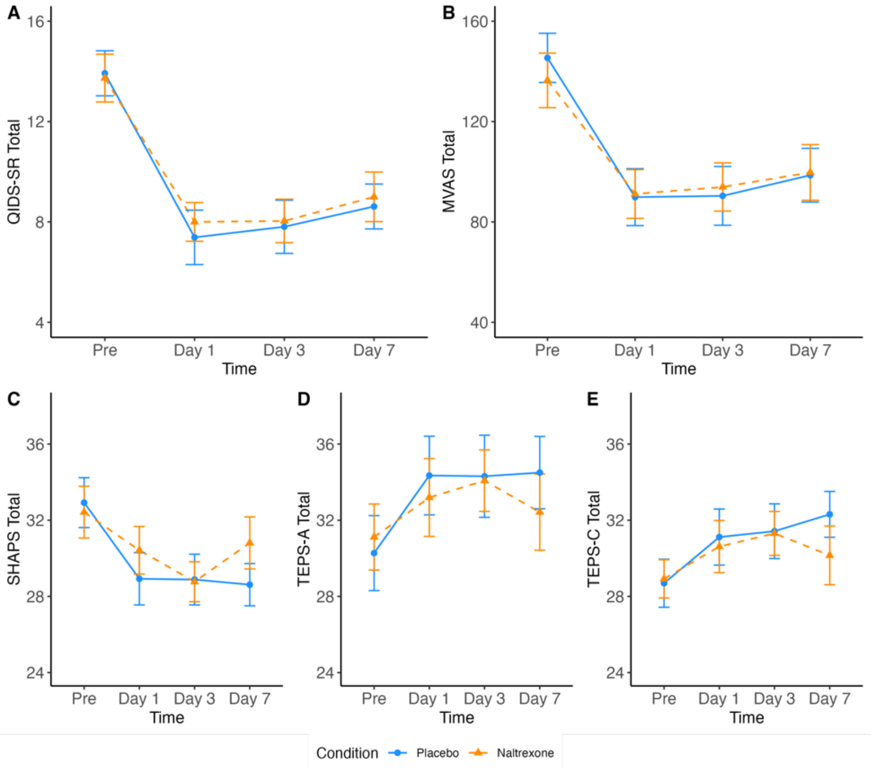

Figure S3: Mean self-report clinical measure scores across post-infusion days 1, 3 and 7: A) QIDS-SR scores; B) M3VAS total scores; C) SHAPS total scores; D) TEPS-A subscale scores (TEPS-A); E) TEPS-C subscale scores. (Error bars ± standard errors). Each plot shows data from n = 26 participants who completed the crossover.

Subgroup analyses of placebo-plus-ketamine condition MADRS and QIDS-SR Day 1 responders

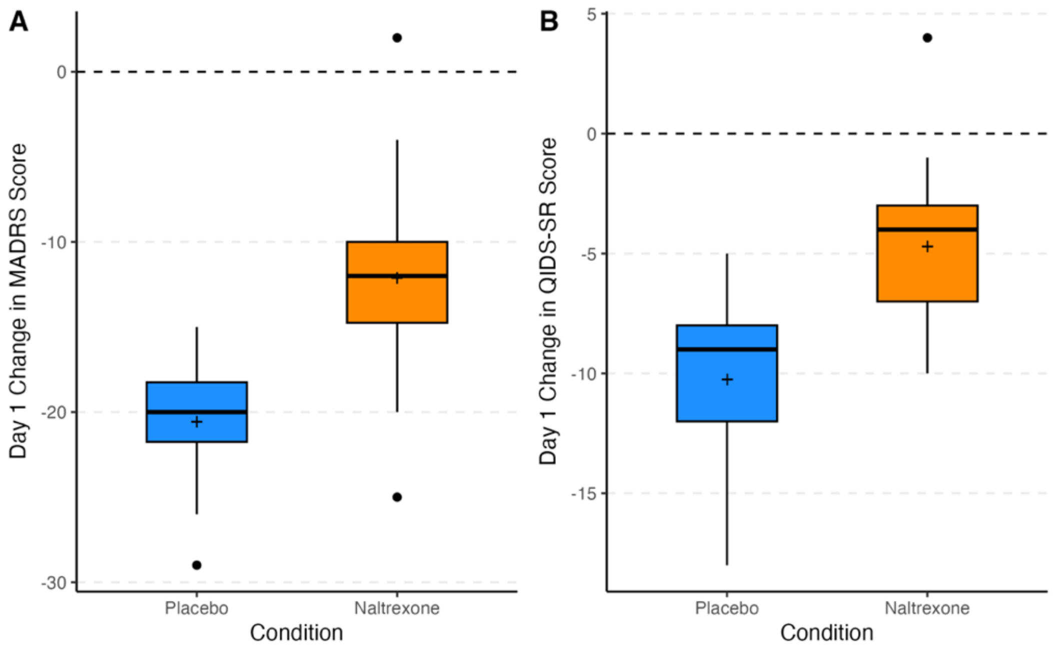

Figure S4: A) Change in MADRS scores from pre-infusion to day 1 post-infusion (Placebo + ketamine MADRS responder subgroup (n = 14)); B) Change in QIDS-SR scores from pre-infusion to day 1 post-infusion (Placebo + ketamine QIDS-SR responder subgroup (n = 13)). Box plot elements: Box spans the interquartile range (IQR) (25th–75th percentile), central line is the median (50th percentile), "+" marks the mean, whiskers extend to the lowest and highest values within 1.5 × IQR of the box, and values beyond that range are shown individually. Each plot shows data from n = 26 participants who completed the crossover.

Of the 26 participants who completed both treatment conditions, 14 met the response criterion of a ≥50% reduction in MADRS score from baseline to day 1 in the placebo-plus-ketamine condition. Additionally, 13 out of the 26 participants met the same response criterion for the QIDS-SR. For the MADRS there was a significant main effect of time ($F_{1,38}$ = 226.90, $p < 0.001$), with reductions in mean MADRS scores at day 1 post-infusion (placebo-plus-ketamine (mean = -20.43, SD = 3.92); naltrexone-plus-ketamine condition (mean= -12.00, SD = 6.62)), and a significantly attenuated reduction for the naltrexone-plus-ketamine condition (mean difference from placebo = 8.43, SD = 8.28, condition-by-time interaction, $F_{1,38}$ = 15.33, p = < 0.001; d = 1.56) (**Supplementary Figure S4A**). For the QIDS-SR there was a significant main effect of time ($F_{1,35}$ = 74.76, $p < 0.001$), with reductions in mean QIDS-SR scores at day 1 post-infusion (placebo-plus-ketamine (mean = -10.15, SD = 3.60); naltrexone-plus-ketamine condition (mean= -4.62, SD = 3.82)), and a significantly attenuated reduction for the naltrexone-plus-ketamine condition (mean difference from placebo = 5.54, SD = 5.70, condition-by-time interaction, $F_{1,35}$ = 10.51, p = 0.003; d = 1.49) (**Supplementary Figure S4B**). Consistent with the primary group analyses, there was a significant main effect of visit on both MADRS ($F_{1,38}$ = 7.50, p = 0.009) and QIDS-SR scores ($F_{1,35}$ = 10.31, p = 0.003).

These exploratory subgroup analyses in identified placebo-plus-ketamine responders, are cautiously presented due to the risks of selection bias and regression to the mean, which may overestimate or misinterpret treatment effects. A future study could address these limitations by first identifying ketamine responders and then randomising them to either placebo-plus-ketamine or naltrexone-plus-ketamine, thereby minimising these biases while preserving the benefits of randomisation.

Clinical and subjective measures by sex

Exploratory linear mixed-effects models examining condition × time × sex interactions for day 1 clinical measure scores revealed no significant effects on MADRS, QIDS-SR, M3VAS, SHAPS, TEPS-A, or TEPS-C (all $p > 0.05$). Although the study was not powered to investigate sex-specific differences, effect size comparisons of change scores between placebo and naltrexone suggested that, in males compared to females, naltrexone produced greater attenuation of symptoms on QIDS-SR, M3VAS, TEPS-A, and TEPS-C (**Supplementary Table S6**).

Exploratory linear mixed-effects models assessing condition × sex interactions for CADSS and PSI subscale item scores revealed no significant effects (all $p > 0.05$).

Table S6: Clinical measure mean day 1 change scores by sex.

Measure	Males			Females		
	**Placebo Day 1 Change Mean (SD)**	**Naltrexone Day 1 Change Mean (SD)**	**Effect Size (D)**	**Placebo Day 1 Change Mean (SD)**	**Naltrexone Day 1 Change Mean (SD)**	**Effect Size (D)**
MADRS	-14.46 (6.23)	-10.54 (7.50)	0.57	-14.85 (9.33)	-10.46 (4.07)	0.60
QIDS-SR	-6.31 (3.86)	-4.92 (3.93)	0.36	-6.77 (6.82)	-6.54 (3.23)	0.04
M3VAS	-58.23 (44.22)	-40.69 (43.77)	0.40	-52.77 (69.46)	-49.85 (51.32)	0.05
SHAPS	-4.23 (6.52)	-2.38 (5.69)	0.30	-3.77 (8.58)	-1.62 (4.31)	0.33
TEPS-A	5.91 (7.69)	2.42 (7.25)	-0.47	2.23 (4.69)	1.74 (7.84)	-0.07
TEPS-C	3.69 (4.52)	2.46 (3.99)	-0.29	1.15 (6.68)	0.92 (4.72)	-0.04

Effect size (D) is for the difference between the placebo and naltrexone conditions.

Clinical measure scores by antidepressant status

Exploratory linear mixed-effects models assessing condition × time × antidepressant status interactions for Day 1 clinical scores revealed no significant effects on MADRS, QIDS-SR, M3VAS, SHAPS, TEPS-A, or TEPS-C (all $p > 0.05$).

MRS checklist, excluded participants and quality metrics

Table S7: MRS Consensus Guidelines Checklist

**Hardware**	
Field strength [T]	3 T
Manufacturer	General Electric
Model (software version if available	MR750
RF coils: nuclei (transmit/receive), number of channels, type, body part	32-channel head coil (Nova Medical Systems)
Additional hardware	None
**Acquisition**	
Pulse sequence	PRESS
Volume of Interest (VOI) locations	Anterior cingulate cortex
Nominal VOI size [cm3, mm3]	20 x 20 x 20mm
Repetition Time (TR), Echo Time (TE) [ms, s]	TR = 2000ms; TE = 40ms
Total number of excitations or acquisitions per spectrum in time series for kinetic studies<br>Number of Averaged spectra per time-point<br>Averaging method (e.g. block-wise or moving average)<br>Total number of spectra (acquired / in time-series)	TOTAL: 1040 acquisitions / 16 water unsuppressed transients<br><br>Block-wise averaging method: Blocks made up of 144 transients
Additional sequence parameters (spectral width in Hz, number of spectral points, frequency offsets)<br>If STEAM: Mixing Time (TM)<br>If MRSI: 2D or 3D, FOV in all directions, matrix size, acceleration factors, sampling method	5000 Hz; 4096 complex points; frequency offset = -2.0 ppm
Water Suppression Method	CHESS
Shimming Method, reference peak, and thresholds for “acceptance of shim” chosen	Automated B0 field mapping
Triggering or motion correction method (respiratory, peripheral, cardiac triggering, incl. device used and delays)	None
**Data analysis methods and outputs**	
Analysis software	FID Appliance (FID-A) toolbox for pre-processing<br>LCModel (Version 6.3-1N)
Processing steps deviating from quoted reference or product	None
Output measure (e.g. absolute concentration, institutional units, ratio)	Metabolite ratios. Referenced to tNAA.
Quantification references and assumptions, fitting model assumptions	Simulated TE = 40 ms basis set containing 19 metabolites
**Data Quality**	
Reported variables (SNR, Linewidth (with reference peaks))	See **Supplementary Table S8**
Data exclusion criteria	FWHM > 0.1 ppm, Cramér-Rao lower bounds (CRLB) for Glx >20%, signal to noise ratio (SNR) <20
Quality measures of postprocessing Model fitting (e.g. CRLB, goodness of fit, SD of residual)	See **Supplementary Table S8**
Sample Spectrum	See **Figure 2**

Excluded participants from ^{1}H-fMRS analysis:

One participant was excluded from the ^{1}H-fMRS analysis due to significant spectral artifacts with severe lipid contamination in one session (**Figure S5**). Another was excluded because the ^{1}H-fMRS sequence was interrupted when the participant requested a pause during scanning in one session.

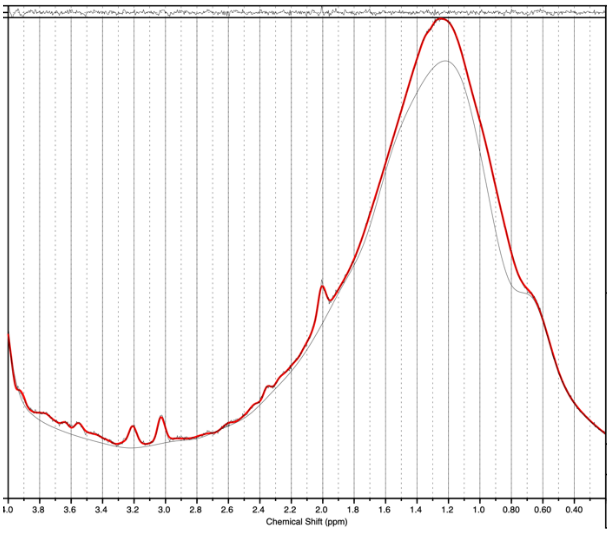

Figure S5: Spectrum from averaged block for excluded participant demonstrating severe lipid contamination. Output of the fit (red) overlaid on the acquired spectrum (black). The estimated baseline is displayed under the spectrum in black.

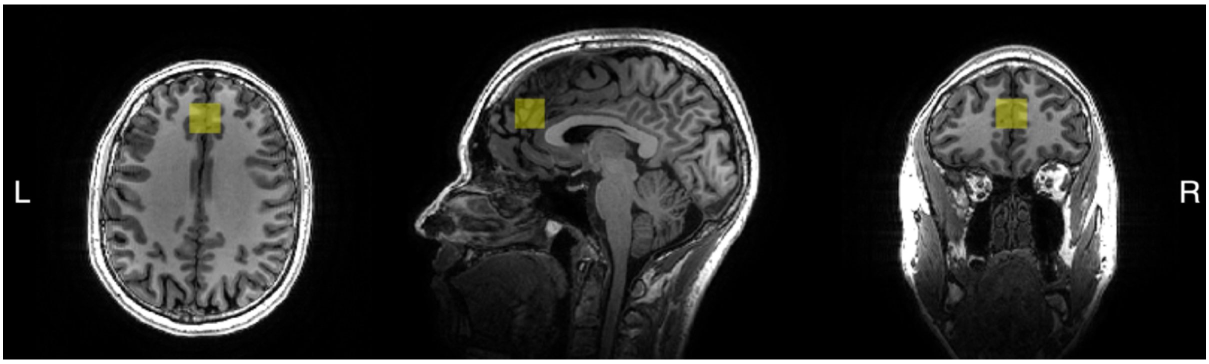

Figure S6: Single subject example placement of ^{1}H-fMRS region of Interest (ROI). ACC Voxel measures 20 (AP) × 20 (RL) × 20 (SI) mm^3 in size. The voxel was prescribed with the centre placed 16 mm above the genu of corpus callosum perpendicular to the AC–PC line.

Table S8: MRS Quality metrics

	Block 1 Mean	Block 1 SD	Block 2 Mean	Block 2 SD	Block 3 Mean	Block 3 SD	Block 4 Mean	Block 4 SD	Block 5 Mean	Block 5 SD	Block 6 Mean	Block 6 SD	Block 7 Mean	Block 7 SD
**CRLB (Glx)**														
**Placebo**	5.250	0.608	5.042	0.550	5.125	0.612	5.250	0.676	5.208	0.658	5.042	0.859	5.167	0.816
**Naltrexone**	5.001	0.659	4.917	0.776	5.167	0.637	5.083	0.717	5.333	0.702	5.042	0.624	5.167	0.482
**FWHM**														
**Placebo**	0.027	0.009	0.029	0.011	0.028	0.013	0.028	0.009	0.027	0.009	0.028	0.007	0.028	0.007
**Naltrexone**	0.026	0.009	0.027	0.008	0.027	0.007	0.026	0.006	0.025	0.006	0.026	0.007	0.027	0.007
**SNR**														
**Placebo**	36.17	6.62	36.17	5.75	35.96	5.72	35.50	6.14	35.75	6.52	35.58	6.45	35.71	6.30
**Naltrexone**	37.83	4.89	36.71	5.33	36.75	4.95	37.13	4.96	37.13	4.60	37.21	4.77	37.33	5.39

Linear mixed effect model analysis revealed a significant main effect of condition ($p < 0.001$) for SNR with higher SNR for the naltrexone (mean = 37.15, SD = 4.92) compared to the placebo condition (mean = 35.83, SD = 6.12). For SNR, there was no significant main effect of block or block-by-condition interactions. For CRLB (Glx) and FWHM there were no significant main effect of block, condition or block-by-condition interactions ($p > 0.05$). CRLB: Cramér Rao Lower Bounds; FWHM: Full Width Half Maximum; SNR: Signal to noise ratio

Table S9: MRS voxel tissue segmentation proportions

	Mean	SD	p-value
**GM**			
Placebo	0.619	0.046	p = 0.250
Naltrexone	0.627	0.051	
**WM**			
Placebo	0.076	0.031	p = 0.625
Naltrexone	0.074	0.031	
**CSF**			
Placebo	0.305	0.056	p = 0.422
Naltrexone	0.299	0.053	

GM: Grey matter, WM: white matter, CSF: cerebrospinal fluid

tCr and tNAA (water referenced)

Linear mixed-effect model analysis revealed a significant main effect of block for tCr change against the pre-infusion baseline ($F_{5,253} = 6.43$, $p < 0.001$), increasing across blocks, and a significant main effect of condition ($F_{1,253} = 12.32$, $p < 0.001$), with higher mean tCr for placebo compared with naltrexone condition and no significant condition-by-block interaction ($F_{5,253} = 0.40$, $p = 0.847$). In contrast, for tNAA change against the pre-infusion baseline there was no significant effect of block ($F_{5,253} = 0.54$, $p = 0.809$), condition ($F_{1,253} = 1.50$, $p = 0.222$), or condition-by-block interaction ($F_{5,253} = 0.08$, $p = 0.995$).

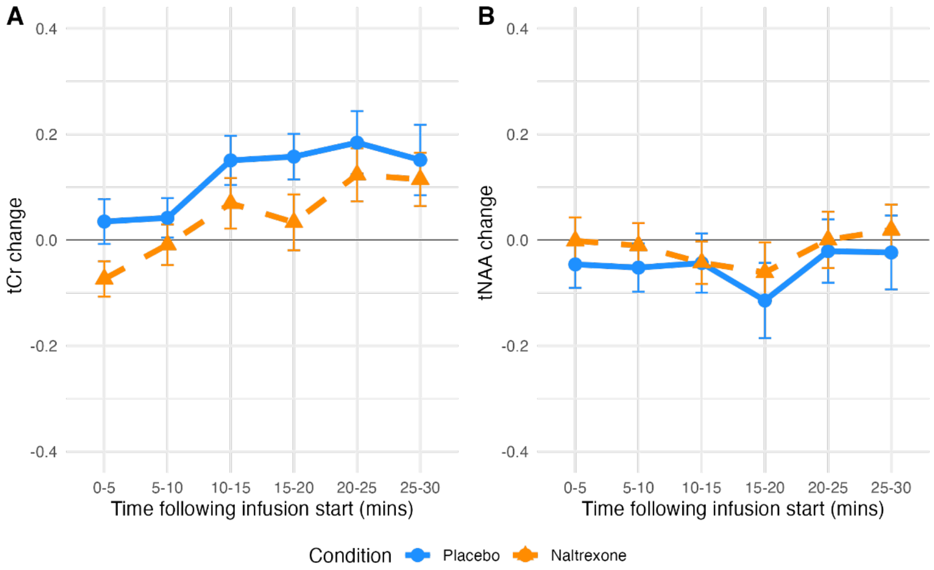

Figure S7: A) Mean tCr change from baseline across ketamine-infusion blocks; B) Mean tNAA change from baseline across ketamine-infusion blocks. (n = 24 participants with complete MRS data passing quality control) (Error bars ± standard errors)

Glx (water referenced)

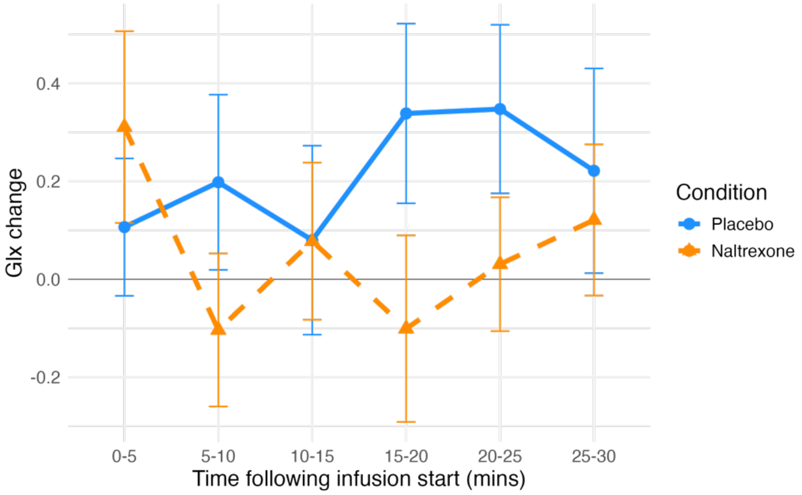

Figure S8: Mean Glx change from baseline across ketamine-infusion blocks. (n = 24 participants with complete MRS data passing quality control) (Error bars ± standard errors)

Glx/tNAA by pre-treatment order

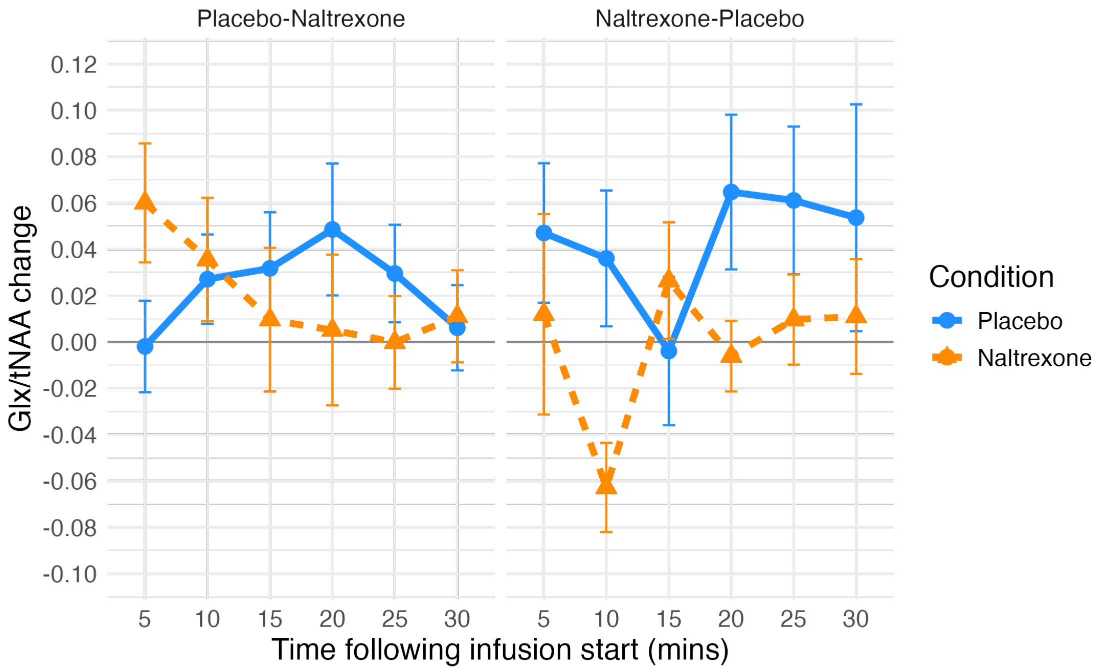

Figure S9: Mean Glx/tNAA change from baseline across ketamine-infusion blocks by pre-treatment order. Placebo-Naltrexone (n = 13) and Naltrexone-Placebo (n = 11) (Error bars ± standard errors)

Glu/tNAA and Gln/tNAA

There were no significant differences in mean baseline Glu/tNAA for the placebo-plus-ketamine condition (mean = 1.06, SD = 0.07) and the naltrexone-plus-ketamine condition (mean = 1.04, SD = 0.08, neither in mean baseline Gln/tNAA for the placebo-plus-ketamine condition (mean = 0.28, SD = 0.06) and the naltrexone-plus-ketamine condition (mean = 0.29, SD = 0.06). Linear mixed-effect model analysis, using the same model parameters as those used for the primary outcome, revealed a significant main effect of condition for Gln/tNAA change against the pre-infusion baseline ($F_{1,253} = 7.49$, $p = 0.007$; $d = 0.38$), with a higher mean increase in Gln/tNAA during the ketamine infusion for placebo compared to the naltrexone pre-treatment condition (**Supplementary Figure S10**). There was no significant main effect of infusion block ($F_{5,253} = 1.17$, $p = 0.324$) or condition-by-block interaction ($F_{5,253} = 0.44$, $p = 0.823$) for Gln/tNAA. For Glu/tNAA there were no significant effects of condition ($F_{1,253} = 0.79$, $p = 0.374$, block ($F_{5,253} = 0.56$, $p = 0.732$) or condition-by-block interaction ($F_{5,253} = 1.55$, $p = 0.176$). Exploratory analyses examining condition-by-sex interactions did not indicate statistically significant effects for Glu/tNAA or Gln/tNAA ($F_{1,262} = 2.65$, $p = 0.10$ and $F_{1,262} = 2.66$, $p = 0.10$ respectively) (**Supplementary Figure S11**).

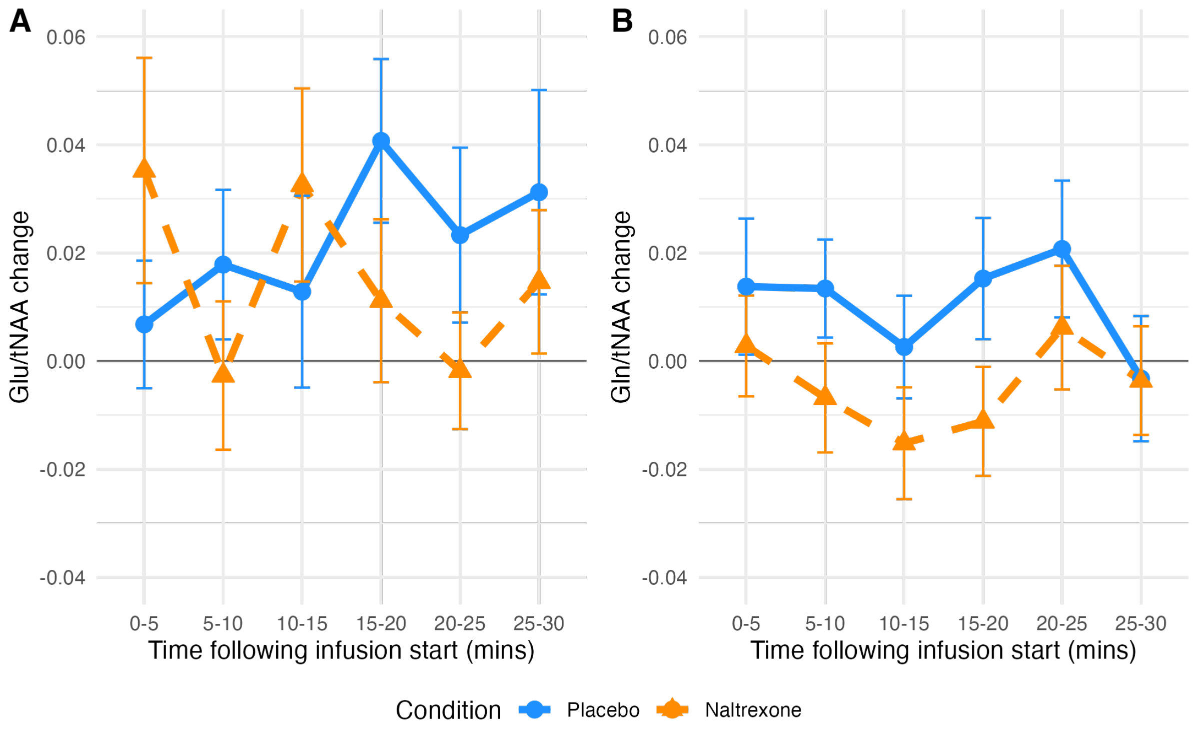

Figure S10: A) Mean Glu/tNAA change from baseline across ketamine-infusion blocks; B) Mean Gln/tNAA change from baseline across ketamine-infusion blocks. (n = 24 participants with complete MRS data passing quality control) (Error bars ± standard errors)

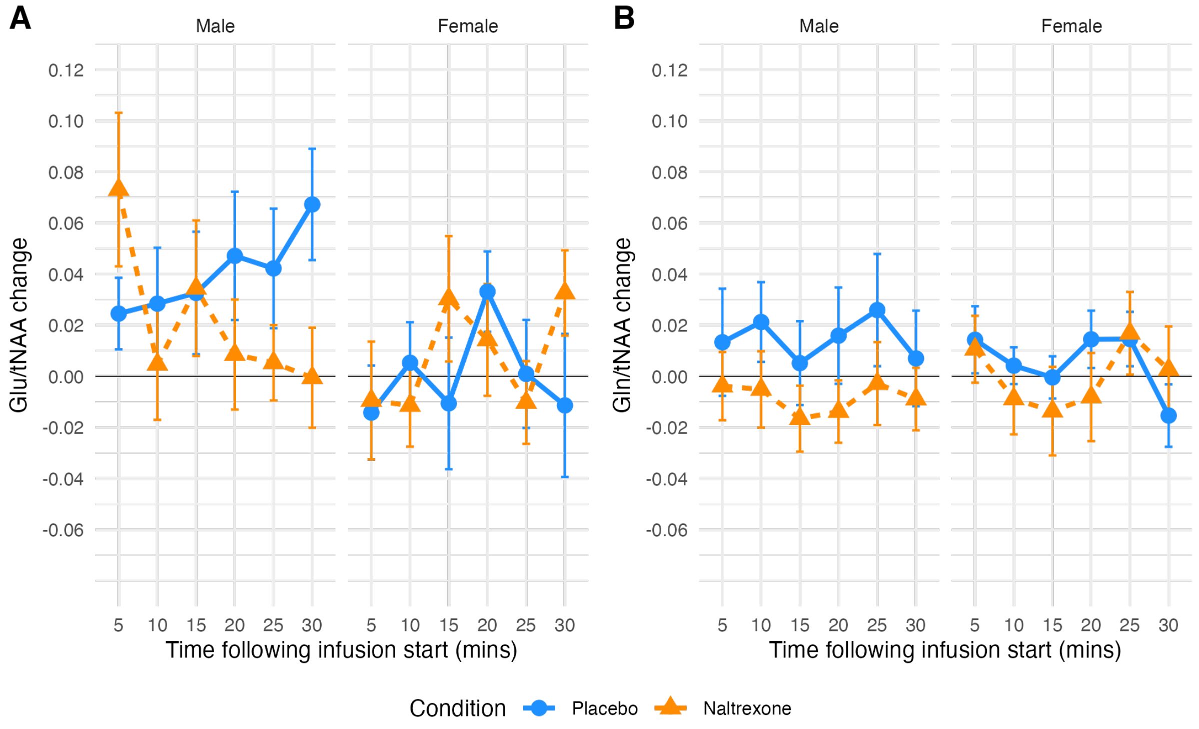

Figure S11: A) Mean Glu/tNAA change from baseline across ketamine-infusion blocks by sex; B) Mean Gln/tNAA change from baseline across ketamine-infusion blocks by sex. Females (n = 11) and males (n = 13) (Error bars ± standard errors)

Glx/tNAA changes and clinical measure correlations

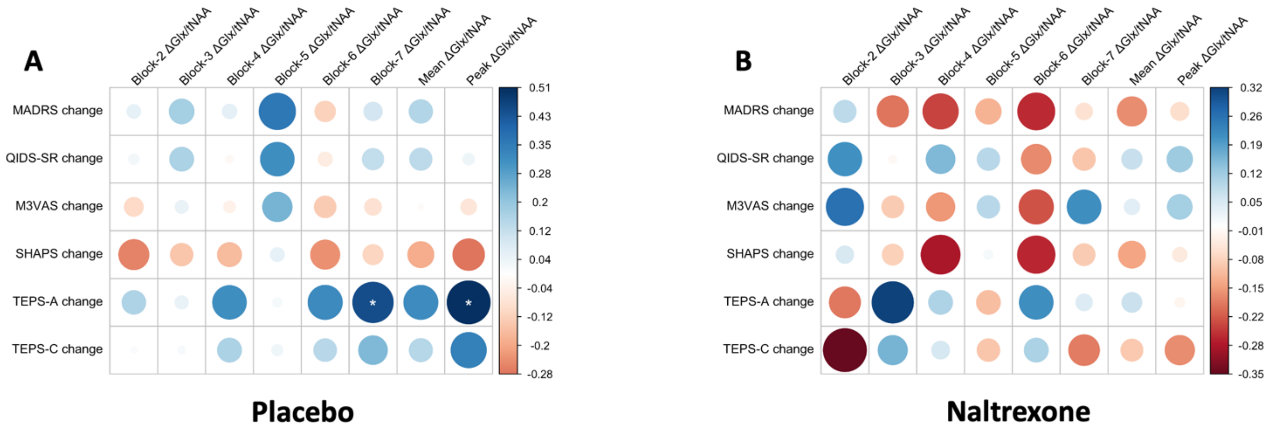

Figure S12: Associations between changes in Glx/tNAA and day 1 clinical measure change scores. A) Correlations between ΔGlx/tNAA from baseline for the six infusion blocks (Blocks 2-7), mean ΔGlx/tNAA across infusion blocks, peak ΔGlx/tNAA and day 1 clinical measure change scores for the placebo pre-treatment condition; B) Correlations between ΔGlx/tNAA from baseline for the six infusion blocks (Blocks 2-7), mean ΔGlx/tNAA across infusion blocks, peak ΔGlx/tNAA and day 1 clinical measure change scores for the naltrexone pre-treatment condition; (*: $p < 0.05$), (**: $p < 0.006$).
