# Supplementary material for: Effect of naltrexone pretreatment on ketamine-induced glutamatergic activity and symptoms of depression: a randomized crossover study
Source: Nat Med. 2025 Jul 24;31(9):2958–66. doi: 10.1038/s41591-025-03800-w (PMC12443602; doi:10.1038/s41591-025-03800-w)
Supplement: Supplementary file 2 — Reporting Summary [file 41591_2025_3800_MOESM2_ESM.pdf]

nature portfolio

Corresponding author(s): Luke Jelen

Last updated by author(s): July 4th 2025

Reporting Summary

Nature Portfolio wishes to improve the reproducibility of the work that we publish. This form provides structure for consistency and transparency in reporting. For further information on Nature Portfolio policies, see our Editorial Policies and the Editorial Policy Checklist.

Statistics

For all statistical analyses, confirm that the following items are present in the figure legend, table legend, main text, or Methods section.

n/a	Confirmed	
☐	☒	The exact sample size (*n*) for each experimental group/condition, given as a discrete number and unit of measurement
☐	☒	A statement on whether measurements were taken from distinct samples or whether the same sample was measured repeatedly
☐	☒	The statistical test(s) used AND whether they are one- or two-sided<br>*Only common tests should be described solely by name; describe more complex techniques in the Methods section.*
☐	☒	A description of all covariates tested
☐	☒	A description of any assumptions or corrections, such as tests of normality and adjustment for multiple comparisons
☐	☒	A full description of the statistical parameters including central tendency (e.g. means) or other basic estimates (e.g. regression coefficient) AND variation (e.g. standard deviation) or associated estimates of uncertainty (e.g. confidence intervals)
☐	☒	For null hypothesis testing, the test statistic (e.g. *F*, *t*, *r*) with confidence intervals, effect sizes, degrees of freedom and *P* value noted<br>*Give P values as exact values whenever suitable.*
☒	☐	For Bayesian analysis, information on the choice of priors and Markov chain Monte Carlo settings
☒	☐	For hierarchical and complex designs, identification of the appropriate level for tests and full reporting of outcomes
☐	☒	Estimates of effect sizes (e.g. Cohen's *d*, Pearson's *r*), indicating how they were calculated

Our web collection on statistics for biologists contains articles on many of the points above.

Software and code

Policy information about availability of computer code

Data collection	Neuroimaging data were collected on a GE Discovery MR750 scanner with software version: "27\\LX\\MR Software release:DV26.0_R03_1831.b". Data were stored and accessed via the centralised Neuroimaging Analysis Network (NaN) at the Centre for Neuroimaging Sciences, Institute of Psychiatry, Psychology and Neuroscience, King's College London. An online randomisation system provided by the King's Clinical Trials unit was used for randomisation and allocation procedures.
Data analysis	Magnetic resonance spectroscopy:<br>- FID Appliance (FID-A) toolbox for preprocessing<br>- LCModel (Version 6.3-1N)<br>Statistical analysis:<br>- R software (version 4.2.1)<br>- nlme package for linear mixed-effects modeling (version 3.1-157)<br><br>R code used for data analysis will be available via the Open Science Framework (OSF) at https://osf.io/96gxt/

For manuscripts utilizing custom algorithms or software that are central to the research but not yet described in published literature, software must be made available to editors and reviewers. We strongly encourage code deposition in a community repository (e.g. GitHub). See the Nature Portfolio guidelines for submitting code & software for further information.

Data

Policy information about availability of data

All manuscripts must include a data availability statement. This statement should provide the following information, where applicable:

- Accession codes, unique identifiers, or web links for publicly available datasets
- A description of any restrictions on data availability
- For clinical datasets or third party data, please ensure that the statement adheres to our policy

De-identified participant data will be accessible upon publication via the Open Science Framework (OSF) at https://osf.io/96gxt/ . All participants have provided consent for their de-identified data to be shared with external entities for scientific research purposes.

Human research participants

Policy information about studies involving human research participants and Sex and Gender in Research.

Reporting on sex and gender	Information on birth sex was collected for demographic characterisation of our sample, based on participant self-report. This data is summarised as numbers and percentages in the demographics table. Exploratory sex-based analyses are reported for the primary outcome.
Population characteristics	The mean age of study participants was 35.1 years. The sample was evenly split by sex (50:50), with 69.2% identifying as White and 65.4% employed. At screening, participants had moderate levels of depression, with a mean Hamilton Depression Rating Scale score of 21.65.
Recruitment	Recruitment was conducted through referrals from primary care and secondary psychiatry services, online advertisements, and word of mouth. Those responding to online advertisements and word of mouth may represent a self-selection bias however the impact of this is mitigated by each participant acting as their own control.
Ethics oversight	Ethical approval was obtained from the London - City & East Research Ethics Committee (Reference: 21/LO/0334).

Note that full information on the approval of the study protocol must also be provided in the manuscript.

Field-specific reporting

Please select the one below that is the best fit for your research. If you are not sure, read the appropriate sections before making your selection.

☒ Life sciences ☐ Behavioural & social sciences ☐ Ecological, evolutionary & environmental sciences

For a reference copy of the document with all sections, see nature.com/documents/nr-reporting-summary-flat.pdf

Life sciences study design

All studies must disclose on these points even when the disclosure is negative.

Sample size	A total of 28 participants were randomised. One participant discontinued after completing one infusion due to adverse events, and another withdrew before the first infusion, stating they no longer wished to participate. Data from 26 participants were available for clinical and subjective measure analysis, and data from 24 participants were available for the primary magnetic resonance spectroscopy analysis.<br><br>Acute ketamine-induced increases in glutamate levels measured using ^{1}H-MRS have previously been reported with medium effect sizes (approximately Cohen's d = 0.75 and Cohen's d = 0.65) [Stone et al., 2012; Javitt et al., 2018]. However, no prior studies have examined the effect of naltrexone on ketamine-induced glutamatergic activity. In our power analysis, it was determined that 24 participants completing both treatment arms were appropriate for detecting a significant difference in glutamatergic measures between the naltrexone and placebo conditions, with a small to medium effect size, using a repeated measures design with an alpha of 0.05 and 80% power.
Data exclusions	Participants who completed both arms of the crossover were included in the primary analyses. Data from 24 participants were available for the primary magnetic resonance spectroscopy analysis after excluding one participant due to significant spectral artifacts and severe lipid contamination in one session (Supplementary Figure S5), and another participant due to an interrupted sequence when they requested a pause during scanning. For the clinical and subjective measure analysis, data from 26 participants were available.
Replication	No replication experiments were conducted because we were only resourced to conduct the core controlled experiment.
Randomization	Participants were assigned to one of two treatment orders—either placebo in the first session followed by naltrexone in the second, or vice versa—using an online randomisation system provided by the King's Clinical Trials Unit. The random sequence was generated using block randomisation with a fixed block size of 4 and stratification by sex to ensure balanced treatment order distribution.
Blinding	The participants and investigators were blinded to treatment assignment. Pharmacy staff, who had access to unblinded treatment assignments, over-encapsulated both the placebo and naltrexone pills to ensure identical appearance and maintain blinding for both

participants and investigators. Unblinding only occurred once all participants had completed both arms of the crossover and data collection was complete.

Reporting for specific materials, systems and methods

We require information from authors about some types of materials, experimental systems and methods used in many studies. Here, indicate whether each material, system or method listed is relevant to your study. If you are not sure if a list item applies to your research, read the appropriate section before selecting a response.

Materials & experimental systems

n/a	Involved in the study
☒	☐ Antibodies
☒	☐ Eukaryotic cell lines
☒	☐ Palaeontology and archaeology
☒	☐ Animals and other organisms
☐	☒ Clinical data
☒	☐ Dual use research of concern

Methods

n/a	Involved in the study
☒	☐ ChIP-seq
☒	☐ Flow cytometry
☐	☒ MRI-based neuroimaging

Clinical data

Policy information about clinical studies

All manuscripts should comply with the ICMJE guidelines for publication of clinical research and a completed CONSORT checklist must be included with all submissions.

Clinical trial registration: NCT04977674

Study protocol: https://clinicaltrials.gov/study/NCT04977674

Data collection: Data was collected by in-person interviews at the NIHR King's Clinical Research Facility, King's College Hospital and by telephone interviews. Participant recruitment and data collection occurred between August 2021 and February 2023.

Outcomes: The primary outcome for the study was the change in Glx (glutamate and glutamine) as measured by magnetic resonance spectroscopy.

Secondary neuroimaging outcomes, as listed (NCT04977674) will be reported separately.

All clinical and subjective outcomes were considered exploratory. For the clinical measures, clinician and self-reported measures of depressive symptoms and anhedonia were measured two hours before each ketamine infusion and at day 1 post-infusion, as previous studies have found antidepressant effects typically peak around 24 hours after a single dose of ketamine. Rating scales included the 10-item Montgomery–Åsberg Depression Rating Scale (MADRS), a clinician-rated measure of depressive severity; the 16-item Quick Inventory of Depressive Symptomatology–Self-Report (QIDS-SR), a self-report instrument covering depressive symptoms; the Maudsley 3-item depression visual analogue scale (M3VAS), a self-rated scale to measure mood, anhedonia and suicidality; the Snaith-Hamilton Pleasure Scale (SHAPS), a 14-item self-rated scale used to measure the state of anhedonia; and the Temporal Experience of Pleasure Scale (TEPS), an 18-item self-rated scale used to assess anticipatory (TEPS-A) and consummatory components (TEPS-C) of pleasure. MADRS response was defined as a reduction from pre-infusion score of ≥ 50% and remission was defined as a MADRS score ≤ 10. QIDS-SR response was defined as a reduction from pre-infusion score of ≥ 50% and remission was defined as a QIDS-SR score ≤ 5. Alongside day 1 post-infusion, self-report measures (QIDS-SR, M3VAS, SHAPS and TEPS) were collected at day 3 and day 7 post-infusion as exploratory outcomes. Subjective effects were measured using the 23-item Clinician Administered Dissociative States Scale (CADSS) and the self-report Psychotomimetic States Inventory (PSI).

Magnetic resonance imaging

Experimental design

Design type: 1H-fMRS block analysis.

Design specifications: 1H-fMRS data were averaged per subject into seven blocks (a baseline block and six blocks during the ketamine infusion), each lasting 288 seconds (144 transients (18 x 8-step phase cycle)).

Behavioral performance measures: No behavioural performance measures were collected during scanning.

Acquisition

Imaging type(s) | 1) Structural and 2) Functional Magnetic Resonance Spectroscopy (1H-fMRS)

Field strength | 3 Tesla

Sequence & imaging parameters

1) STRUCTURAL
A high-resolution sagittal T1-weighted (T1-w) 3D sagittal inversion recovery prepared spoiled gradient echo (IR-SPGR) scan was initially acquired for localisation of the spectroscopy voxel (TR = 7.35 ms, TE = 3.04 ms, TI = 400 ms, FOV = 270 mm, flip-angle = 11°, matrix size = 256 × 256, slice thickness = 1.2 mm, 196 slices).

2) 1H-fMRS
Spectra were acquired continuously for a 5 minute 'baseline' period and during the initial 30 minutes of the ketamine infusion using Point RESolved Spectroscopy (PRESS), with CHEmical Selective Suppression for water suppression and outer volume suppression (OVS) with Very Selective Suppression (VSS) pulses (TR = 2,000 ms, TE = 40 ms, 8-step phase cycle, 1040 transients/16 water unsuppressed transients).

Further details are provided in the minimum reporting standards for in vivo magnetic resonance spectroscopy checklist (Supplementary Table S7), according to consensus recommendations.

Area of acquisition

The 1H-fMRS voxel was positioned in an ACC region-of-interest (20mm×20mm×20mm) with the centre of the voxel placed 16 mm above the most anterior portion of the genus of the corpus callosum, perpendicular to the anterior commissure- posterior commissure line to minimise inclusion of white matter and cerebral spinal fluid (CSF) (Supplementary Figure S6).

Diffusion MRI ☐ Used ☒ Not used

Preprocessing

Preprocessing software | FID Appliance (FID-A) toolbox for preprocessing [Near et al. 2015, Simpson et al. 2017]

Normalization

To assess the consistency of MRS voxel tissue composition, the voxels were co-registered to the T1-weighted image and segmented using the Gannet CoRegStandAlone function, which calls SPM12 to estimate the voxel fraction of grey matter (GM), white matter (WM) and cerebrospinal fluid (CSF) for each condition. The MRS voxels were spatially normalised to the MNI152 template image using the SPM12 spatial normalisation function. This process involved applying the deformation fields derived from the T1-weighted images to which each MRS voxel had been co-registered. Subsequently, each voxel was converted to a binary mask using FSL. These binary masks were then combined to illustrate the positional overlap among participants and across conditions.

Normalization template | The MRS voxels were spatially normalised to the MNI152 template image using the SPM12 spatial normalisation function.

Noise and artifact removal

Data were pre-processed using an automated FID-A pipeline, which includes coil combination, removal of motion corrupted scans and spectral registration for frequency and phase drift correction [Near et al. 2015, Simpson et al. 2017]. If motion corrupted scans were identified, the relevant transients were removed before determining the affected block average, ensuring the start time of each block remained consistent across participants and conditions.

Volume censoring | As above.

Statistical modeling & inference

Model type and settings

A linear mixed-effects model for repeated measures was used for the primary outcome, Glx/tNAA change from baseline. The following fixed effects were included in the model: condition (placebo and naltrexone), block (six ketamine infusion blocks) and the interaction between condition and block. Participant ID was included as a random intercept.

Effect(s) tested

Tested the main effect of pre-treatment condition (placebo and naltrexone), block (six ketamine infusion blocks) and the interaction between condition and block on Glx/tNAA change from baseline.

Specify type of analysis: ☐ Whole brain ☒ ROI-based ☐ Both

Anatomical location(s)

The 1H-fMRS voxel was positioned in an ACC region-of-interest (20mm×20mm×20mm) with the centre of the voxel placed 16 mm above the most anterior portion of the genus of the corpus callosum, perpendicular to the anterior commissure- posterior commissure line to minimise inclusion of white matter and cerebral spinal fluid (CSF) (Supplementary Figure S6).

Statistic type for inference
(See Eklund et al. 2016)

This was not an fMRI study, and no voxel-wise or cluster-wise statistics were used for inferences.

Correction

This was a single region-of-interest magnetic resonance spectroscopy voxel, and no correction for multiple comparisons was applied.

Models & analysis

n/a	Involved in the study
☒	☐ Functional and/or effective connectivity
☒	☐ Graph analysis
☒	☐ Multivariate modeling or predictive analysis
